# G protein-coupled KISS1 receptor is overexpressed in triple negative breast cancer and promotes drug resistance

**DOI:** 10.1038/srep46525

**Published:** 2017-04-19

**Authors:** Alexandra Blake, Magdalena Dragan, Rommel G. Tirona, Daniel B. Hardy, Muriel Brackstone, Alan B. Tuck, Andy V. Babwah, Moshmi Bhattacharya

**Affiliations:** 1 Department of Physiology and Pharmacology, Schulich School of Medicine and Dentistry, The University of Western Ontario, London, ON, Canada; 2 Division of Clinical Pharmacology, Department of Medicine, Schulich School of Medicine and Dentistry, The University of Western Ontario, London, ON, Canada; 3 Lawson Health Research Institute, London, ON, Canada; 4 Department of Obstetrics and Gynecology, Schulich School of Medicine and Dentistry, The University of Western Ontario, London, ON, Canada; 5 Department of Oncology, Schulich School of Medicine and Dentistry, The University of Western Ontario, London, ON, Canada; 6 Division of Surgical Oncology, Schulich School of Medicine and Dentistry, The University of Western Ontario, London, ON, Canada; 7 Department of Pathology, Schulich School of Medicine and Dentistry, The University of Western Ontario, London, ON, Canada; 8 The Pamela Greenaway-Kohlmeier Translational Breast Cancer Research Unit, London Regional Cancer Program, London, Ontario, Canada; 9 The Children’s Health Research Institute, London, ON, Canada.

## Abstract

Triple-negative breast cancer (TNBC) lacks the expression of estrogen receptor α, progesterone receptor and human epidermal growth factor receptor 2 (HER2). TNBC patients lack targeted therapies, as they fail to respond to endocrine and anti-HER2 therapy. Prognosis for this aggressive cancer subtype is poor and survival is limited due to the development of resistance to available chemotherapies and resultant metastases. The mechanisms regulating tumor resistance are poorly understood. Here we demonstrate that the G protein-coupled kisspeptin receptor (KISS1R) promotes drug resistance in TNBC cells. KISS1R binds kisspeptins, peptide products of the *KISS1* gene and in numerous cancers, this signaling pathway plays anti-metastatic roles. However, in TNBC, KISS1R promotes tumor invasion. We show that *KISS1* and *KISS1R* mRNA and KISS1R protein are upregulated in TNBC tumors, compared to normal breast tissue. KISS1R signaling promotes drug resistance by increasing the expression of efflux drug transporter, breast cancer resistance protein (BCRP) and by inducing the activity and transcription of the receptor tyrosine kinase, AXL. *BCRP* and *AXL* transcripts are elevated in TNBC tumors, compared to normal breast, and TNBC tumors expressing KISS1R also express AXL and BCRP. Thus, KISS1R represents a potentially novel therapeutic target to restore drug sensitivity in TNBC patients.

Breast cancer is the leading cause of cancer related deaths in women worldwide^1^. Triple-negative breast cancer (TNBC) comprises of 15–20% of breast cancers, occurring often in women under 50 years of age^2^. TNBC tumors lack estrogen receptor α (ERα), progesterone receptor (PR) and human epidermal growth factor receptor 2 (HER2 or ErbB2)^2^. TNBC patients lack targeted therapies and have the worst prognosis compared to patients with other breast cancer subtypes. This is often due to high rates of metastases and disease recurrence after an initial response to standard chemotherapy (eg anthracyclines, taxanes), because the tumors become drug resistant^3^. Moreover, these patients have limited treatment options^4^,^5^ and thus there is a dire need for understanding the biological pathways that are distinctly activated in TNBC, that could lead to the development of better targeted therapies. G protein-coupled receptors (GPCRs), targets for 50% of current pharmaceutical agents, are critical players in tumor metastasis^6^, yet very little is known about their roles in TNBC or in regulating tumor drug resistance.

KISS1R (aka GPR54), a Gα_q/11_-coupled GPCR, is a key regulator of the reproductive axis. Kisspeptins (KPs), products of the *KISS1* gene, bind and activate KISS1R^7^,^8^,^9^. KPs (10, 13, 14 and 54 amino acids) are secreted, biologically active, blood-borne peptides, derived from a pro-peptide KISS1, that is cleaved rapidly by matrix metalloproteinases (MMPs), MT1-MMP1, MMP-9 and furin, to form KP-10^7^,^10^,^11^, a highly studied peptide also produced by breast cancer cells^12^,^13^,^14^,^15^,^16^. *KISS1* and *KISS1R* mRNA are expressed at several sites throughout the body including the normal breast^7^,^11^,^17^,^18^,^19^. Although *KISS1* (commonly classified as a metastasis suppressor gene) exerts anti-cancer roles^20^ and loss of *KISS1* correlates with poor patient prognosis in many cancers^21^,^22^,^23^, KISS1R appears to play a pro-metastatic role in some cancers such as breast and liver cancers^20^,^24^. For example, once breast cells lose ERα, KISS1R signaling appears to become detrimental, promoting epithelial to mesenchymal transition (EMT)^18^ and cell invasion, by stimulating invadopodia formation (*via* MT1-MMP^25^). KISS1R can also stimulate TNBC invasion by activating the epidermal growth factor receptor (EGFR, HER1), and stimulating MMP-9 secretion and activity^16^,^18^. We and others have demonstrated that ERα negatively regulates *KISS1* mRNA levels^19^ as well as KISS1R-induced invasion^18^ in breast cancer. This may partially account for why KP/KISS1R promotes metastasis in TNBC, where ERα is absent. ERα also negatively regulates *KISS1* expression in the arcuate nucleus of the hypothalamus^26^. In support of these observations in breast cancer, treatment of ERα-positive breast cells with tamoxifen (an ER antagonist commonly used as an anti-cancer drug), increased *KISS1* and *KISS1R* mRNA levels^19^. These findings were further supported by a report that women with ERα-positive tumors who were treated with tamoxifen exhibited high *KISS1* and *KISS1R* mRNA levels, which were associated with poor prognosis^19^. Other studies found that *KISS1* and *KISS1R* mRNA and protein levels are higher in ERα-negative invasive ductal carcinoma, than ERα-positive primary tumors and this correlated with poor patient outcome^27^,^28^,^29^. More recently, in a mouse mammary tumor virus model, *Kiss1r* has been shown to stimulate breast cancer metastasis^30^. However, whether KISS1 or KISS1R are expressed in TNBC tumors and promotes drug resistance in TNBC is unknown and being investigated in this study.

Treatment for TNBC patients is currently limited to surgery and conventional chemotherapies, including anthracyclines such as doxorubicin that is considered to be one of the most effective agents to treat TNBC^31^. Initially patients respond well, however they often develop chemoresistance that is a main cause of poor outcome in many TNBC patients^32^. Resistance to current standard regimens limits the available options for previously treated patients. Although the underlying mechanisms are poorly understood, studies have implicated a role for increased survival factors or inactivation of cell death pathways in regulating TNBC drug resistance^33^. Drug resistance is also mediated by multidrug resistance proteins such as ATP-binding cassette (ABC) transporters which facilitate the efflux of various substrates across cell membranes, including anti-cancer drugs^3^,^32^. Among the forty-nine human ABC transporters, three are well-known for regulating chemoresistance to doxorubicin namely, ABCB1/p-glycoprotein (P-gp), ABCC1/multidrug resistance-associated protein 1 (MRP 1), and ABCG2/breast cancer resistance protein (BCRP)^32^. Among these, BCRP is highly expressed in TNBC tumors^34^. Although several inhibitors of ABC transporters have been tested clinically, the clinical trials have not been successful^3^,^32^. Another key regulator of drug resistance that has emerged is the receptor tyrosine kinase AXL, a transforming oncogene^35^ that is also highly expressed in TNBC primary tumors and TNBC cell lines^36^. AXL, a member of the Tyro, AXL and Mer (TAM) receptor family of receptor tyrosine kinases is a key regulator of EMT as well as drug resistance to doxorubicin and anti-EGFR therapies in many cancer types^37^,^38^. In TNBC patients, high levels of AXL expression correlate with a significant decrease in patient survival^36^.

Here, we demonstrate for the first time that *KISS1* and *KISS1R* mRNA and KISS1R protein levels are upregulated in primary TNBC tumors, compared to normal breast and that KISS1R signaling promotes drug resistance in multiple ERα-negative breast cell lines and in TNBC cells by stimulating the expression of BCRP, as well as by promoting *AXL* gene transcription and activity. Furthermore, we demonstrate that antagonizing KISS1R resensitizes tumor cells to chemotherapy.

## Methods

### Cell Culture

Cells were purchased from ATCC and maintained at 37 °C with 5% CO_2_. MCF10A cells were grown in mammary epithelial basal medium (Clonetics-Cambrex) supplemented with a MEGM Single Quots kit and cholera toxin (100 ng/mL). SKBR3 and MDA-MB-231 were cultured in RPMI 1640 supplemented with 10% (v/v) fetal bovine serum (FBS). Cell lines stably expressing KISS1R (MCF10AFLAG-KISS1R and SKBR3FLAG-KISS1R) and pFLAG vector controls were generated as described^18^ and represent polyclonal cell populations; these were grown in media containing G418 (1.5 ng/mL) and proteins expression verified weekly by Western blot analysis.

### Quantitative real-time PCR and Origene human TNBC cDNA panels

Total RNA was extracted from cells using the RNeasy Mini Kit (Qiagen). Reverse-transcription was carried out according to manufacturer’s instructions using iScript RT Supermix (Bio-Rad). Gene expression was determined using SYBR green real-time qPCR (RT-qPCR) as previously described^39^. *KISS1R, KISS1, AXL* and *BCRP* transcript levels in primary TNBC tumors and normal breast tissue were determined using TissueScan™ Disease Tissue qPCR Human Breast Cancer Arrays (OriGene Technologies, Rockville MD). Each array comprised of 20 TNBC breast tumors and 10 normal breast tissue samples, accompanied by pathologist report (Table 1). These panels with the accompanying β-actin controls contain de-identified human TNBC patient samples at various stages of cancer development. The steady-state mRNA levels of each gene of interest were determined by amplification of cDNA using specific primers: *KISS1* forward primer (F) 5′-GGACCTGCCTCTTCTCACCA-3′ and reverse primer (R) 5′-ATTCTAGCTGCTGGCCTGTG-3′; *KISS1R* (F): 5′-CCCACCCTCTGGACATTCAC-3′ and (R): 5′CCTAGAAGTGCCTTGAGGCTTG-3′; *AXL* (F): 5′-CAGCAAGAGCGATGTGTGGT-3′ and (R): 5′-CGATTTCCCTGGCGCAGATA-3′; *BCRP* (F): 5′-TGGCTGTCATGGCTTCAGTA-3′ and (R): 5′-GCCACGTGATTCTTCCACAA-3′; *GADPH* (F): 5′-TGGTATCGTGGAAGGACTCA-3′ and (R): 5′-TTCAGCTCAGGGATGACCTT-3′. Results were normalized to *GAPDH*. Statistical analysis of gene expression in clinical samples were conducted by a biostatistician (Statistical Services, Western) using Wilcoxon two-sample test.

### MTT Assay

MTT cell viability assays were conducted as previously described^39^ (Cell Signaling)^39^. Briefly, 7.5 × 10^4^ cells were plated in a 96-well plate and treated with 1 μM P-234 or vehicle overnight in media containing 10% FBS. Prior to doxorubicin treatment, cells were pretreated with a cocktail of drug efflux transporter inhibitors for 10 minutes: 50 μM MK-571 (Alexis Biochemicals), 1 μM Fumitremorgin C (Sigma), 1 μM Zosuquidar (Lilly Laboratories) or vehicle. A dose-response experiment was conducted in the presence of the inhibitors, using graded concentrations of doxorubicin, ranging from 0.01 μM to 300 μM (MCF10A and SKBR3 cells) or 0.01 μM to 2 mM (MDA-MB-231 cells), for 48 hours. Media was then aspirated and cells were incubated with 0.5 mg/mL of MTT labeling (3-(4,5-dimethylthiazol-2-yl)-2,5-diephenyltetrazolium bromide) agent for 4 h and subsequently solubilized with DMSO. Absorbance was read at 550 nm with a background subtraction at 670 nm, using a Victor V_3_ plate reader (Perkin Elmer).

### Doxorubicin Accumulation Assay

Cells were treated with doxorubicin (1 μM, 2 hours) as described^40^. Cells were fixed with 4% paraformaldehyde (room temperature, 20 minutes) and washed with Hank’s Balanced Salt Solution (HBSS). Nuclei were then stained with 0.01% Hoechst 33258 (Invitrogen). Cells were mounted on slides and imaged using an LSM-510 META laser scanning microscope (Zeiss, Germany).

### Immunoblot Assays

Experiments were performed as described^16^,^18^. Cells were lysed using RIPA buffer and protein separated by SDS-PAGE. Protein expression was examined using antibodies raised against human proteins: rabbit anti-KISS1R (1:4000, Abcam), rabbit anti-KISS1 (1:500; Abcam), rat anti-BCRP (1:150, Abcam), rabbit anti-cleaved-PARP (1: 1000, Cell Signaling), rabbit anti-PARP (1: 1000, Cell Signaling), mouse anti-Histone H3 (1:1000; Millipore); mouse anti-HSP90 (1:1000, Cell Signaling); mouse anti-phosphoptyrosine (PY-20, 1:1000, Millipore); rabbit anti-AXL (1:2000, Cell Signaling), rabbit anti-snail/slug (1:500, Abcam), mouse anti-N-cadherin (1:500, BD Biosciences), rabbit anti-ERK (1:1000, Cell Signaling), rabbit anti-phoshpo-ERK1/2 (1:2000, Cell Signaling), rabbit anti-AKT (1:1000, Cell Signaling), rabbit anti-phospho-AKT (1:1000, Cell Signaling), rabbit anti-survivin (1:1000, ThermoScientific), rabbit anti-SP-1 (1:1000, Millipore), mouse anti-tubulin (1:5000, Abcam) or mouse anti-β-actin (1:1000, ThermoScientific), rabbit anti-β-actin (1:2000, ThermoScientific), GADPH (1:2000, Abcam) and visualized by chemiluminescence. β-actin, GADPH or tubulin expression was used as a loading control.

For human breast protein analysis, immunoblots were conducted using TNBC biopsies obtained from Dr. Brackstone’s London Tumor Biobank (Table 2), in accordance with the Health Sciences Research Ethics Board at the University of Western Ontario. Core tumor tissues (10 mm × 1 mm) were collected by guided needle biopsy and immediately frozen in liquid nitrogen and the diagnosis was confirmed by the pathologist. Normal breast tissue (non-cancerous) was also obtained from the London Tumor Biobank. The cores were homogenized in RIPA lysis buffer containing proteases inhibitors, sonicated and centrifuged at 4 °C and protein expression in 100 μg lysates was analyzed by Western blot analysis^16^,^18^. The relative expression of KISS1 or KISS1R to GADPH was also normalized to the expression of KISS1 or KISS1R in MDA-MB-231 cell lysates (20 μg). MDA-MB-231 protein expression therefore served as an internal control in all studies; lysates from the same lot were used.

### Human TNBC Tumor immunostaining

These experiments were conducted as previously described^41^. Formalin-fixed, paraffin-embedded human TNBC tumor blocks were reviewed by pathologist (Dr A. Tuck, London Health Sciences Center) and studies conducted in accordance with the Ethics Board at the University of Western Ontario. Sections (5 μm thick) were de-paraffined, cleared, rehydrated and followed by heat induced antigen retrieval and auto-fluorescence quenching (1 mg/ml sodium borohydride in TBS) prior to immunostaining. KISS1R rabbit polyclonal antibody (1:50; Abcam), AXL goat polyclonal antibody (1:20; R&D), BCRP mouse polyclonal (1:20; Abcam) and KISS1 rabbit polyclonal (1:200; Abcam) were incubated overnight followed by donkey anti-rabbit AF488 (1:250; Invitrogen), donkey anti-goat AF555 (1:500; Invitrogen) and Hoechst (1:10000; Invitrogen) staining. Slides were imaged using Zeiss LSM800 laser scanning microscope.

### KP Secretion Assay

MDA-MB-231 and SKBR3FLAG-KISS1R cells were grown at a density of 5 × 10^5^ cells/well. The SKBR3pFLAG vector control cells, which grow slowly were plated at a density of 1 × 10^6^ cells/well. Next day, cells were washed and incubated in complete media (phenol red-free RPMI with 10% FBS) for 24 hrs. The conditioned media was then collected, cleared by centrifugation (5 min, 300 g) and 50 μl was used to measure secreted kisspeptin levels using the Fluorescent enzyme immunoassay (EIA) kit: Human KISS1 (68–121) Amide/Metastin (1–54) from Phoenix Pharmaceuticals Inc (CA), according to manufacturer’s instructions. This EIA measures all forms of kisspeptins. FBS used in complete media contained kisspeptin at a concentration of 2.3 ± 0.6 pmol/L and was therefore subtracted from all readings. Secretion was normalized to the protein concentration of cells in each well.

### Scratch Assays for Cell Motility

These assays were done as previously described^18^,^25^. Scratch closure by cells in FBS supplemented media was done using an automated Olympus IX-81 microscope. Cells migrating into the scratch over 24 hours were imaged every 15 minutes. Distance travelled was quantified using an *In Vivo* Analyzer Suite (Media Cybernetics; Rockville, MD, USA) and this software was used for time-lapse microscopy (see Supplemental Movie). For each image (per time point), the width of the scratch (μm) was measured at seven points along the scratch. The distance migrated was calculated by subtracting the width of the scratch at each time point from the width of the scratch at time zero. The distances migrated into the scratch at each of the seven points/image was averaged to determine the distance migrated for each well.

### AXL Depletion by siRNA

SKBR3FLAG-KISS1R cells were grown to 60% confluency and transfected with 100 pmol of control or AXL siRNA (Ambion, Life Technologies) using jetPRIME reagent, according to the manufacture’s instructions (Polyplus Transfection). AXL knock-down was determined by RT-qPCR and Western blot analysis.

### Chromatin immunoprecipitation (ChIP)

ChIP assay was performed using EZ-Magna ChIP^TM^ G-Chromatin Immunoprecipitation Kit (Millipore) according to the manufacturer’s protocol. Briefly, DNA was cross-linked to protein (1% formaldehyde, 20 minutes). Unreacted formaldehyde was then quenched with 137.5 mM glycine for 5 minutes at room temperature. Cells were then lysed and sonicated to shear DNA to a length of 200 to 1,000 base pairs. Chromatin samples were then incubated with 20 μL protein G magnetic beads and antibodies to SP-1 (4 μg/sample, Millipore), RNA polymerase II (RNA Pol II, 2 μg/sample, Millipore) or non-immune IgG from the same host species (anti-rabbit or anti-mouse, Millipore). After overnight incubation, beads were washed, samples were reverse crosslinked and chromatin was eluted with Elution Buffer and Proteinase K treatment. DNA was subsequently purified using spin columns. The binding of SP-1 and RNA Pol II to the human BCRP and AXL promoters was quantified by RT-qPCR. For BCRP, we used (F) 5′-CAATGAGCGCCTGGTGATTCT-3′ and (R) 5′-GGTCACCCTGCCGTGACA-3′ primers that amplify105 bp region surrounding the two distal SP-1 binding sites on the *BCRP* promoter (−278 to −173) involved in regulating basal BCRP gene expression and chemoresistance^42^. For AXL, RT-qPCR was employed using (F) 5′-TTGAGTTAACCCCTGATTGTCCAG-3′ and (R) 5′-CTCACTCCCAGACTTGGGCA-3′ primers that amplify a 107 bp region surrounding the two proximal SP-1 binding sites on the *AXL* promoter (−298 to −189) implicated in regulating constitutive *AXL* gene expression^43^. Using serial dilutions of human chromosomal DNA, these primers were demonstrated to have equal efficiency in priming their target sequences.

## Results

### *KISS1R* and *KISS1* transcripts are upregulated in human primary TNBC tumors

**Figure 1:**
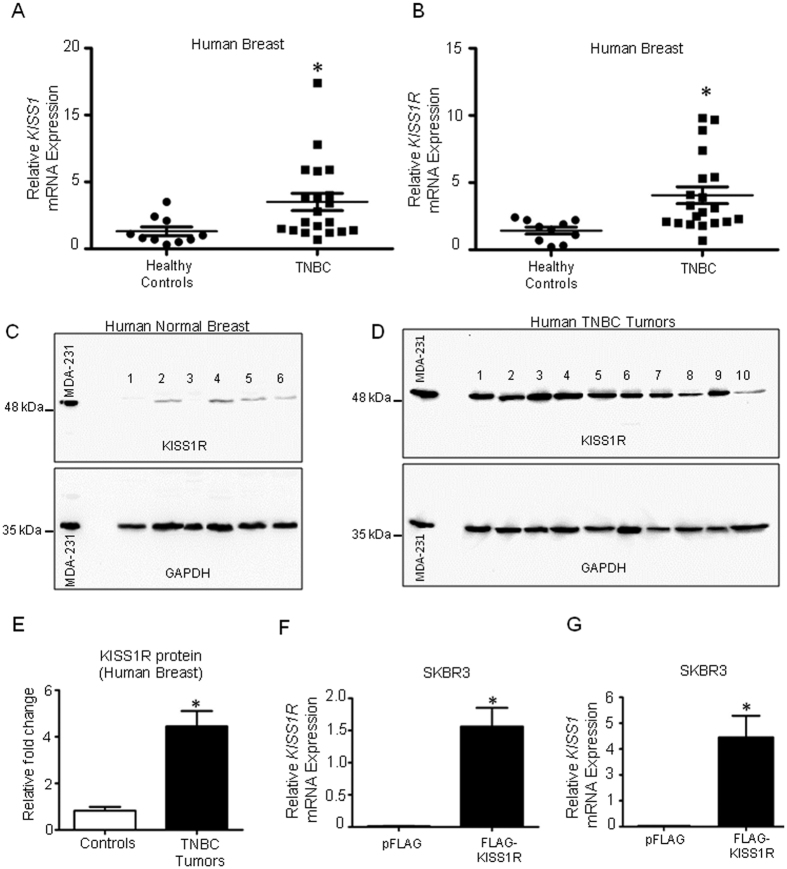
KISS1 and KISS1R expression is elevated in TNBC primary tumors. Scatter plots of mRNA levels for (**A**) *KISS1* (**B**) *KISS1R* in 20 TNBC primary breast tumor tissues and 10 normal breast tissue (Origene cDNA array), as determined by qPCR. Samples were normalized to β-actin expression. Non-parametric Wilcoxon Two-Sample Test was applied to all expression data. *P = 0.008 for *KISS1R*, 0.006 for *KISS1*. (**C**,**D**) Representative western blots showing the expression of endogenous KISS1R in breast tissue from 13 healthy subjects and 20 TNBC primary tumors, relative to expression of each protein in MDA-MB-231 cell lysates (positive control); see Supplemental Fig. 1 for remaining blots. (**E**) Densitometric analysis of blots conducted by normalizing band intensity to GADPH loading controls and protein expression in MDA-MB-231 cell lysates (internal control). (**F**,**G**) Relative mRNA expression of *KISS1R* and *KISS1* by RT-qPCR in SKBR3 cells stably expressing KISS1R and pFLAG vector controls. Columns represent mean relative mRNA expression, normalized to GAPDH ± SEM; student’s unpaired T-test: *P < 0.05. (n = 4).

Although clinical data show that *KISS1* (which encodes kisspeptins) and *KISS1R* are more strongly expressed in ERα-negative primary tumors, compared to normal breast^19^,^27^,^29^, expression of *KISS1* and *KISS1R* specifically in TNBC is unknown. We therefore quantified *KISS1* and *KISS1R* expression in a TNBC cDNA array (Origene) and found that the mRNA levels of both genes were significantly greater in 20 TNBC primary breast tumors, compared to 10 normal breast tissue biopsies (Fig. 1A,B; p = 0.006 for KISS1 and 0.008 for KISS1R, Table 1). Western blot analysis revealed that KISS1R protein was elevated in TNBC primary tumors compared to normal breast (Fig. 1C–E, Supplementary Fig. 1A,C, Table 2). However, KISS1 protein could not be detected in these tissue samples using this method. Since we were able to detect exogenous kisspeptin-54 and endogenous KISS1 in MDA-MB-231 cell lysates (Supplementary Fig. 1B,D), we conclude that KISS1 levels were too low to be detected in these tumor and normal breast samples.

**Figure 2:**
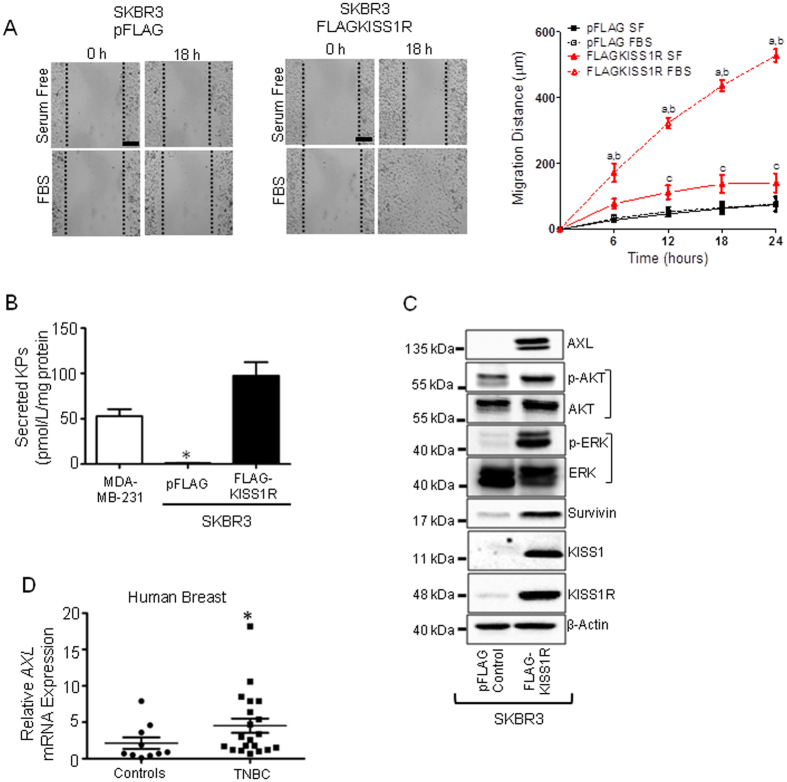
KISS1R signaling increases cell motility, kisspeptin secretion and pro-survival molecules. (**A**) KISS1R overexpression in SKBR3 cells increases the distance closed by FLAG-KISS1R cells compared to pFLAG controls over 24 hour period (n = 3). Two-way ANOVA followed by Bonferroni post-hoc test: a, P < 0.05 for pFLAG FBS vs FLAG-KISS1R FBS; b, P < 0.05 for FLAG-KISS1R SF vs FLAG-KISS1R FBS; c, P < 0.05 for FLAG-KISS1R SF vs pFLAG SF. *Scale Bar*, 100 μm. (**B**) Secreted kisspeptin levels in conditioned media from cells, measured using a Fluorescent EIA human KISS1 assay. One-way ANOVA followed by Bonferroni post-hoc test; P < 0.05. (**C**) Representative western blots (cropped) showing KISS1R overexpression in SKBR3FLAG-KISS1R and controls and the endogenous expression of KISS1 peptide, AXL, ERK and AKT (phosphorylated and total) and survivin. See Supplementary Fig. 3 for quantification of blots (n = 4–6) and Supplementary Fig. 8A–F for full blots. (**D**) Scatter plots of *AXL* mRNA levels in 20 TNBC primary breast tumor tissues and 10 normal breast tissue (Origene cDNA array), as determined by real-time PCR. Samples were normalized to β-actin expression. Non-parametric Wilcoxon Two-Sample Test was applied to all expression data. *P = 0.008 for *AXL*.

TNBC cells such as MDA-MB-231 and Hs578T cells express endogenous KISS1R^25^, and we have previously reported that KISS1R signaling in these cells stimulates cell invasion by stimulating invadopodia formation^25^. We also used two ERα-negative cell lines, normal (non-malignant) MCF10A and breast cancer SKBR3 cells that we previously reported express very low levels of endogenous KISS1R^18^. Consequently, these cell lines were used for stably expressing FLAG-KISS1R^18^. We have also shown that KISS1R over-expression in these two *gain-of-function* models promoted an EMT-like event, resulting in increased tumor cell migration and invasion^18^, hallmarks of metastasis. SKBR3FLAG-KISS1R cells not only displayed increased *KISS1R* mRNA and protein levels (Figs 1F and 2C, Supplementary Fig. 3A) but also exhibited higher levels of KISS1 mRNA and protein compared to pFLAG vector controls (Figs 1G and 2C, Supplementary Fig. 3B). Interestingly, using a scratch/wound healing assay, the expression of FLAG-KISS1R in SKBR3 cells (Supplementary Fig. 1E) stimulated cell motility and scratch closure over 24 h, compared to pFLAG vector controls, (Fig. 2A, Supplemental Movie; Supplementary Fig. 1F). To exclude possibility that scratch closure and increased KP secretion were due to increased cell proliferation resulting from KISS1R overexpression, cell growth assays were performed and revealed that both SKBR3pFLAG and SKBR3FLAG-KISS1R had doubling time of over 24h (Supplemental Figure 2). Interestingly, SKBR3FLAG-KISS1R secreted kisspeptins to a greater extent than pFLAG controls or TNBC MDA-MB-231 cells (Fig. 2B). Taken together, we conclude that the effects of KISS1R overexpression on scratch closure and kisspeptin secretion was not due to increased cell proliferation of SKBR3 cells.

**Figure 3:**
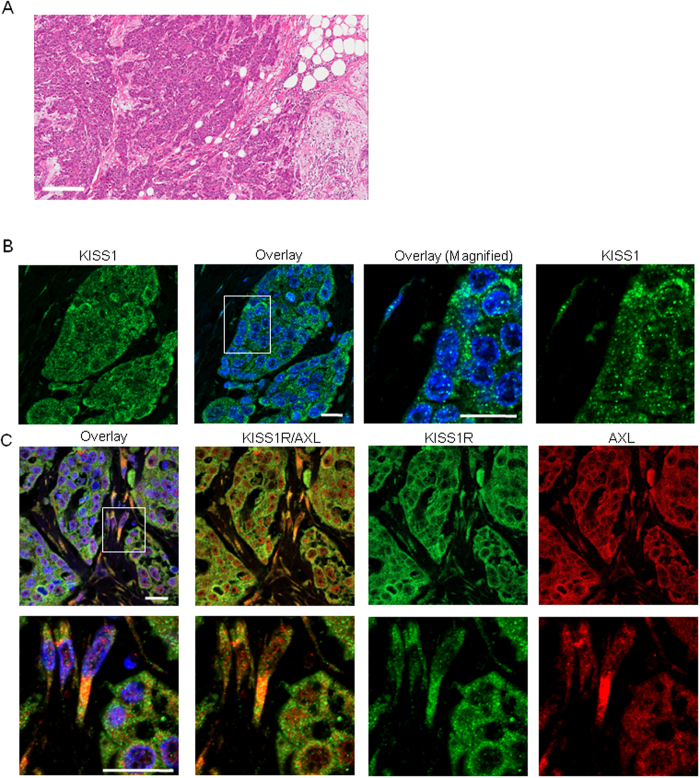
KISS1 and KISS1R localization in TNBC patient tumors. (**A**) Representative image of TNBC tumor tissue stained with hematoxylin and eosin, showing invasive ductal carcinoma. *Scale Bar*, 200 μm. Representative images obtained from immunofluorescent confocal microscopy of the same TNBC patient tumor sections using (**B**) rabbit polyclonal KISS1 followed by donkey anti-rabbit AF488 (1:250; Invitrogen) (**C**) rabbit polyclonal KISS1R and goat polyclonal AXL antibodies followed by donkey anti-rabbit AF488 (green) or donkey anti-goat AF555 (red). Hoechst (blue) for nuclear stain. Boxed area (upper panel) shows magnified area in lower panel. Yellow (in overlay) indicates areas of co-localization of AXL (red) and KISS1R (green). *Scale Bar*, 20 μm.

We also observed a significant increase in the the expression of pro-survival molecules AXL, AKT, ERK, as well as the anti-apoptotic protein, survivin upon over-expression of KISS1R in SKBR3 cells (Fig. 2C, Supplementary Fig. 3C–F). As previously reported^36^, we found that *AXL* mRNA levels were significantly increased in TNBC patients, compared to normal breast tissue (Fig. 2D, p = 0.033). In the TNBC samples, *AXL* expression correlated positively with *KISS1* expression (r = 0.549, p = 0.012 by Spearman rank correlation); a significant correlation between AXL or KISS1 with KISS1R or BCRP was not observed in this TNBC cohort. We also performed immunofluorescence analysis of patient tumors to determine whether these proteins localized to primary TNBC tumors (Fig. 3). Results revealed a punctate distribution of KISS1 (Fig. 3B) and a robust localization of KISS1R and AXL in tumor cells (Fig. 3C). Partial co-localization (yellow, in confocal image overlay) was also observed in tumors and stromal cells (Fig. 3C, lower panel). Overall, the data clearly show that *KISS1, KISS1R* and *AXL* are aberrantly expressed in human TNBC tumors. Since AXL signaling promotes chemoresistance^44^, we therefore hypothesize that KISS1R signaling via AXL confers drug resistance in TNBC patients.

### KISS1R signaling promotes drug resistance

**Figure 4:**
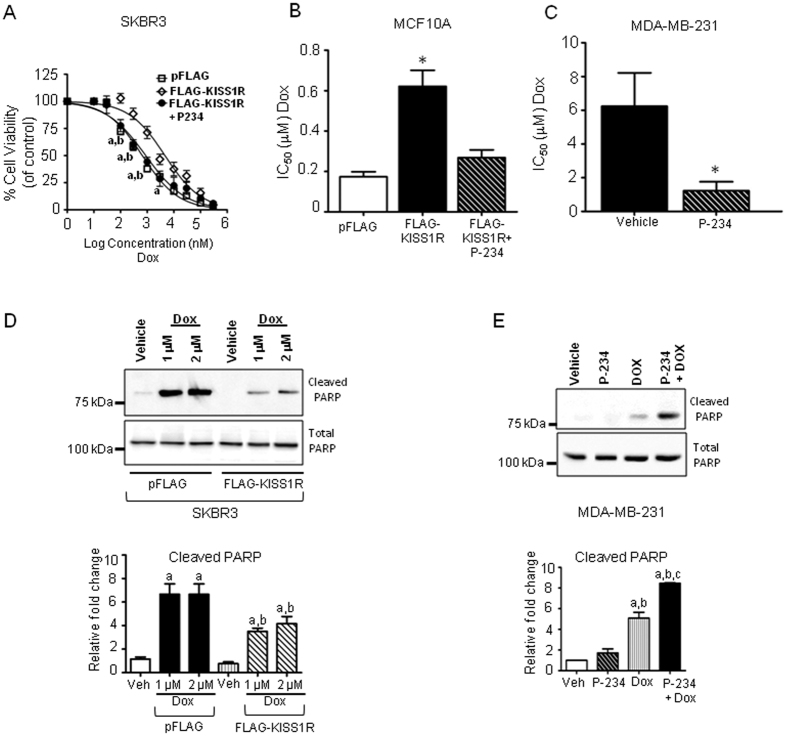
KISS1R signaling induces drug resistance in ERα-negative breast cells and TNBC cells. (**A**–**C**) MTT cell viability assays of cells grown in graded concentrations of Dox; cells pretreated with KISS1R antagonist, P-234 (1 μM, overnight). Cell viability normalized to matching vehicle concentrations (DMSO). For A, Two-way ANOVA followed by a Bonferonni post hoc test: a, P < 0.05, pFLAG vs FLAGKISS1R; b, P < 0.05, FLAGKISS1R + P-234 vs FLAGKISS1R; For B-C: inhibitory concentration (IC_50_) values calculated for each individual curve (n = 5–7); one-way ANOVA followed by Dunnet’s multiple comparison test (for **A**) or student’s unpaired T-test (for C): *P < 0.05. (**D**,**E**) Representative western blots (cropped) showing cleaved and total PARP in (**D**) SKBR3FLAG-KISS1R cells and controls treated with doxorubicin (Dox, 1 μM or 2 μM, 48 h); (**E**) TNBC MDA-MB-231 cells treated with Dox (2 μM, 48 h), in the presence or absence of P-234 (1 μM, overnight). Densitometric analysis of blots shown below of cleaved PARP, normalized to total PARP in each cell line (n = 3). For D: one-way ANOVA followed by Bonferroni’s multiple comparison test. a, P < 0.05, Veh vs. Dox; b, P < 0.05, FLAG-KISS1R (Dox) vs pFLAG control (Dox). For **E**: one-way ANOVA followed by Bonferroni’s multiple comparison test. a, P < 0.05, Veh vs. Dox; b, P < 0.05, P-234 vs Dox; c, P < 0.05 for Dox vs. P-234 + Dox. See Supplementary Fig. 8G,H for full-length blots.

The ability of cancer cells to become resistant to chemotherapies is a major obstacle in treating cancer patients and results in poor patient outcome^45^. Since cell survival molecules were highly expressed in SKBR3FLAG-KISS1R cells compared to pFLAG vector controls, we used these cells to initially evaluate a role of KISS1R in regulating the drug resistant phenotype by conducting MTT cell viability assays in the presence of graded concentrations of doxorubicin, an anthracycline used in adjuvant TNBC chemotherapy^31^. We found that SKBR3FLAG-KISS1R cells displayed increased cell survival in the presence of doxorubicin, compared to vector controls (Fig. 4A).

P-234 is an established KISS1R antagonist and has been used to block KISS1R activity in several *in vivo* and *in vitro* systems^18^,^25^,^46^,^47^,^48^,^49^. When SKBR3FLAG-KISS1R cells were treated P-234, the dose response curve shifted to the left, indicating increased cell death, similar to the response observed in controls (Fig. 4A). P-234 increased drug sensitivity in MCF10AFLAG-KISS1R and metastatic TNBC MDA-MB-231 cells (Fig. 4B,C) which express endogenous KISS1R^25^.

During apoptosis, poly (ADP-ribose) polymerase (PARP) is cleaved by caspases to generate an 89 kDa fragment from the 116 kDa full-length protein. To confirm that KISS1R expression conferred resistance to apoptosis, we examined the expression of cleaved PARP in SKBR3FLAG-KISS1R cells, following treatment with doxorubicin (1 and 2 μM) or vehicle for 48 hours. We observed that for both doxorubicin concentrations, cleaved PARP was significantly greater in control cells than in SKBR3FLAG-KISS1R cells (Fig. 4D). Similarly, treatment of TNBC MDA-MB-231 cells with doxorubicin (2 μM, 48 hrs) increased cleaved PARP, and this was further increased following P-234 treatment (Fig. 4E). Collectively, these results indicate that KISS1R signaling promotes tumor chemoresistance and pharmacological inhibition of KISS1R re-sensitizes cancer cells to doxorubicin, enabling them to undergo apoptosis.

### KISS1R signaling promotes the expression of drug efflux transporter BCRP

**Figure 5:**
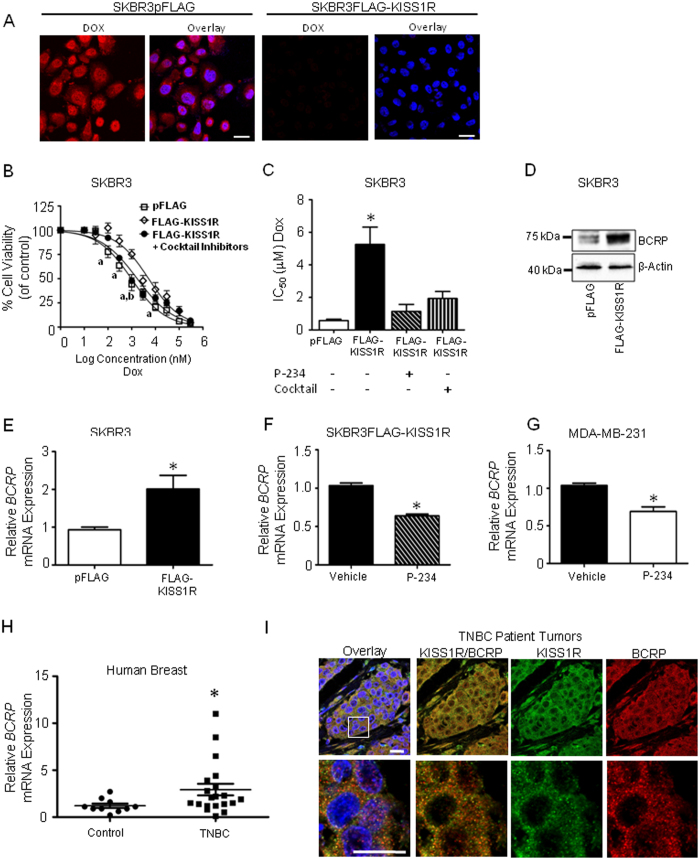
KISS1R induces the expression of drug transporter BCRP. (**A**) Representative confocal images showing KISS1R expression confers drug resistance, decreasing the intracellular accumulation of doxorubicin (DOX, 1 μM, 2h) in cells. (red, DOX autofluorescence; blue: Hoeschst nuclear stain). Scale bars, 20 μm; n = 4. (**B**,**C**) MTT cell viability assays; cells treated with a cocktail of drug efflux transporter inhibitors (50 μM MK-571, 1 μM Fumitremorgin C, 1 μM Zosuquidar, 10 min), P-234 (1 μM, overnight) or vehicle (n = 6). Cell viability normalized to matching vehicle (DMSO). Two-way ANOVA followed by a Bonferonni post hoc test: a, P < 0.05 for pFLAG vs FLAG-KISS1R; b, p < 0.05 for FLAG-KISS1R (Cocktail) vs FLAG-KISS1R (vehicle). (**C**) IC_50_ was calculated for each individual curve; one-way ANOVA followed by Dunnet’s multiple comparison test: *P < 0.05 (n = 6). (**D**) Representative western blot (cropped) showing the endogenous expression of efflux drug transporter BCRP in SKBR3FLAG-KISS1R cells and vector controls (n = 5); see Supplementary Fig. 3G for quantification of blots and Supplementary Fig. 8I for full-length blots. (**E**) *BCRP* mRNA expression in SKBR3FLAG-KISS1R cells and controls (n = 4). (**F**,**G**) P-234 pretreatment decreased *BCRP* mRNA expression; cells were treated with 1 μM P-234 or vehicle control in serum-free conditions for 48 hours; qPCR performed using primers for BCRP and GAPDH (n = 3). Student’s unpaired T-test: *P < 0.05. Columns represent mean relative mRNA expression ± SEM, normalized to GAPDH. (**H**) Scatter plot of *BCRP* mRNA levels in 20 TNBC primary breast tumor tissues compared to 10 normal breast tissue (Origene cDNA array), as determined by qPCR. Samples were normalized to β-actin expression. Non-parametric Wilcoxon Two-Sample Test was applied to all expression data; *P = 0.03. (**I**) Representative images showing immunofluorescence and confocal microscopy of patient TNBC tumor sections using rabbit polyclonal KISS1R followed by donkey anti-rabbit AF488 (green) and mouse polyclonal BCRP antibodies, followed by donkey anti-mouse AF555 (red). Hoechst (blue), to stain nuclei. Boxed area (upper panel) shows magnified area in lower panel. Yellow (overlay) indicates areas of co-localization of proteins. *Scale Bar*, 20 μm.

Intracellular drug accumulation and efficacy of chemotherapeutic agents are dependent in part on the interplay between cellular drug uptake and efflux processes. We therefore assessed whether KISS1R expression reduced the cellular accumulation of chemotherapeutic doxorubicin^31^, a compound that autofluoresces red and is a substrate for some ABC transporters including BCRP^40^,^50^. Doxorubicin accumulation (Fig. 5A) was dramatically reduced in SKBR3FLAG-KISS1R cells, compared to control cells, suggesting a role for KISS1R-dependent doxorubicin efflux as a mechanism of drug resistance. Thus, to investigate a role of ABC transporters in regulating KISS1R-induced drug resistance, we examined the effect of pretreating SKBR3FLAG-KISS1R cells and controls with a cocktail of efflux drug transporters inhibitors: Fumitremorgin C (BCRP inhibitor), MK-571 (MRP inhibitor) and Zosuquidar (P-gp inhibitor) and found that cell viability decreased to levels observed in controls (Fig. 5B). SKBR3FLAG-KISS1R cells had significantly higher inhibitory concentration (IC_50_) values when compared to pFLAG cells, and treatment of SKBR3FLAG-KISS1R cells with the KISS1R antagonist or drug efflux transporter inhibitors restored cell sensitivity to doxorubicin (Fig. 5C).

Studies have demonstrated that doxorubicin is a substrate for drug efflux transporter BCRP, and overexpression of BCRP is associated with increased drug resistance in TNBC^34^,^51^. We found that KISS1R overexpression in SKBR3 cells resulted in higher expression levels of BCRP mRNA and protein levels, compared to controls (Fig. 5D,E, Supplementary Fig. 3G), whereas pharmacological inhibition of KISS1R using P-234 decreased *BCRP* mRNA levels (Fig. 5F). This finding was recapitulated in MDA-MB-231 cells, where P-234 treatment resulted in a decrease in *BCRP* mRNA levels (Fig. 5G). In TNBC patients, *BCRP* mRNA levels were significantly increased compared to normal breast (Fig. 5H, p = 0.029). We also found that cells expressing KISS1R in TNBC tumor biopsies also expressed BCRP (Fig. 5I). Taken together, these results indicate that KISS1R signaling in breast cancer promotes doxorubicin resistance by upregulating BCRP expression, leading to reduced intracellular drug accumulation and reduced cytotoxicity.

### KISS1R signaling stimulates expression and activity of the pro-survival molecule AXL

**Figure 6:**
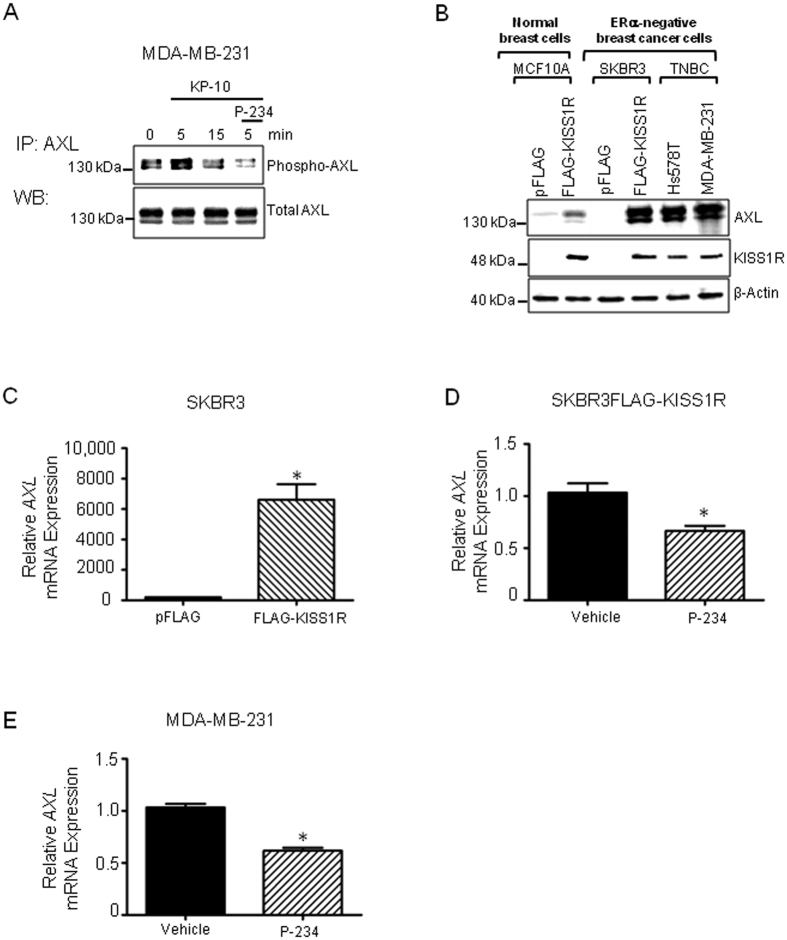
KISS1R induces AXL activity and expression. (**A**) Representative blot (cropped) showing KP-10 (100 nM) stimulates AXL phosphorylation in TNBC MDA-MB-231 cells; pretreatment of cells with KISS1R antagonist, P234 (1 μM, overnight), inhibits KP-10 induced phosphorylation of AXL (n = 4). AXL immunoprecipitated with a rabbit anti-AXL and blotted with anti-PY20 antibody to detect phosphorylated tyrosines. (**B**) AXL protein expression in mammary cell lysates. KISS1R overexpression in *normal* breast MCF10A cells or in SKBR3 induces AXL expression (n = 5). (**C**) *AXL* mRNA expression in SKBR3FLAG-KISS1R cells and controls (n = 5). (**D**,**E**) P-234 decreased *AXL* mRNA expression in breast cancer cells; cells were treated with 1 μM P-234 (48 h) or vehicle in serum-free conditions. Total RNA was isolated from cells and RT-qPCR performed using primers for AXL and GAPDH (n = 3). Student’s unpaired T-test: *P < 0.05. Columns represent mean relative mRNA expression, normalized to GAPDH ± SEM. See Supplementary Fig. 4A,B for the densitometric analysis of blots and Supplementary Fig. 8J for full-length blots.

Up-regulation of pro-survival pathways can lead to drug resistance^52^. Since KISS1R induced AXL expression (Fig. 2C), we investigated whether KISS1R signaling also stimulated AXL activation. Stimulation of TNBC MDA-MB-231 cells with KP-10 (100 nM)^16^,^18^ induced a transient but rapid and robust activation of AXL which was inhibited by the KISS1R antagonist, P-234 (Fig. 6A, Supplementary Fig. 4A). We previously showed that KISS1R expression triggered AXL expression in the ERα-negative cells SKBR3 breast cancer cells, which normally do not express AXL (Fig. 2C). We now demonstrate that this increase in AXL protein expression (Fig. 6B, Supplementary Fig. 4) is likely due to increased *AXL* expression (Fig. 6C). P-234 treatment of SKBR3FLAG-KISS1R or MDA-MB-231 cells down-regulated *AXL* mRNA (Fig. 6D,E). KISS1R also increased AXL levels in ERα-negative, normal, MCF10A breast cells (Fig. 6B, Supplementary Fig. 4B). Furthermore, we found that the TNBC cell lines (MDA-MB-231 and Hs578T) which endogenously express KISS1R also robustly express AXL (Fig. 6B), but the ERα-positive breast cancer cells (T47D and MCF7), that barely express KISS1R, do not express AXL (Supplementary Fig. 5). Taken together, this data suggests that KISS1, KISS1R and AXL are upregulated in invasive breast cancer cells (Hs578T, MDA-MB-231, SKBR3FLAG-KISS1R), compared to non or weakly invasive breast cancer cells (SKBR3, MCF7 and T47D) (Fig. 6B; Supplementary Figs 4B and 5).

### KISS1R promotes drug resistance via AXL

**Figure 7:**
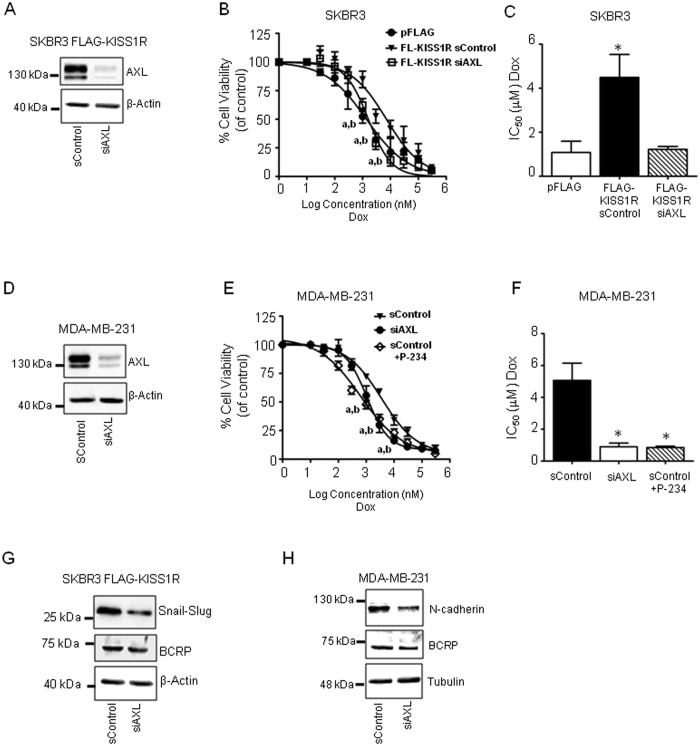
KISS1R regulates drug resistance via AXL. (**A**) Representative Western blot (cropped) showing protein levels of AXL in SKBR3FLAG-KISS1R cells expressing AXL siRNA and scrambled controls, 48 h after transfection (n = 5). (**B**) SKBR3FLAG-KISS1R cells expressing AXL siRNA or scrambled were treated with graded concentrations of doxorubicin. Cell viability by MTT assay was normalized to matching vehicle concentrations (n = 4). Two-way ANOVA followed by a Bonferonni post hoc test: a, P < 0.05 for pFLAG vs FLAG-KISS1R Scrambled; b, P < 0.05 for FLAG-KISS1R siAXL vs FLAG-KISS1R Scrambled. (**C**) IC_50_ values calculated for each individual curve from B; n = 4. One-way ANOVA followed by Dunnet’s multiple comparison test: *P < 0.05. (**D**) Representative Western blot (cropped) showing AXL protein expression in TNBC MDA-MB-231 cells expressing AXL siRNA and scrambled control, 48 h after transfection (n = 3). (**E**) MTT cell viability assays of MDA-MB-231 cells upon AXL depletion and (**F**) IC_50_ values calculated for each individual curve from **E**. Scrambled controls were treated with P-234 (1 μM, overnight) or vehicle; cell viability was normalized to matching vehicle concentrations (n = 4). One-way ANOVA followed by Dunnet’s multiple comparison test: *P < 0.05. (**G**,**H**) Representative Western blot showing protein levels of EMT markers (snail-slug, N-cadherin) and BCRP in SKBR3FLAG-KISS1R or MDA-MB-231 cells expressing AXL siRNA and scrambled controls, 48 h after transfection (n = 3). See Supplementary Fig. 4C–H for the quantification of blots and Supplementary Fig. 8K–O for full-length blots.

Since KISS1R signaling significantly increased AXL expression (Fig. 6B) and activity (Fig. 6A), and AXL has been shown to promote TNBC drug resistance^37^,^38^, a role for AXL in KISS1R-induced drug resistance was investigated. To do this, AXL was depleted in ERα-negative cells and TNBC cells using siRNA. AXL depletion in SKBR3FLAG-KISS1R cells or TNBC MDA-MB-231 cells (Fig. 7A,D, Supplementary Fig. 4C,D) restored doxorubicin sensitivity in both cell lines (Fig. 7B,E), as reflected by significantly lower IC_50_ values, compared to scrambled controls (Fig. 7C,F). Moreover, IC_50_ values in AXL-depleted SKBR3FLAG-KISS1R cells approached values similar to vector controls (Fig. 7C). Similarly, a reduction in IC_50_ for doxorubicin cytotoxicity was demonstrated in AXL-depleted MDA-MB-231 cells to level similar to that found in cells treated with P-234 (Fig. 7F). We also observed that depletion of AXL resulted in reduced levels in EMT marker snail-slug in SKBR3FLAG-KISS1R cells and N-cadherin in MDA-MB-231 cells (Fig. 7G,H; Supplementary Fig. 4E,G); the expression of BCRP however, remained unchanged (Fig. 7G,H; Supplementary Fig. 4F,H) as long as 96 h post transfection with AXL siRNA (Supplementary Fig. 6). Overall, these findings suggest that KISS1R signals via AXL to promote chemoresistance through a mechanism that may be independent of BCRP upregulation.

### KISS1R regulates *AXL* transcription via SP-1

**Figure 8:**
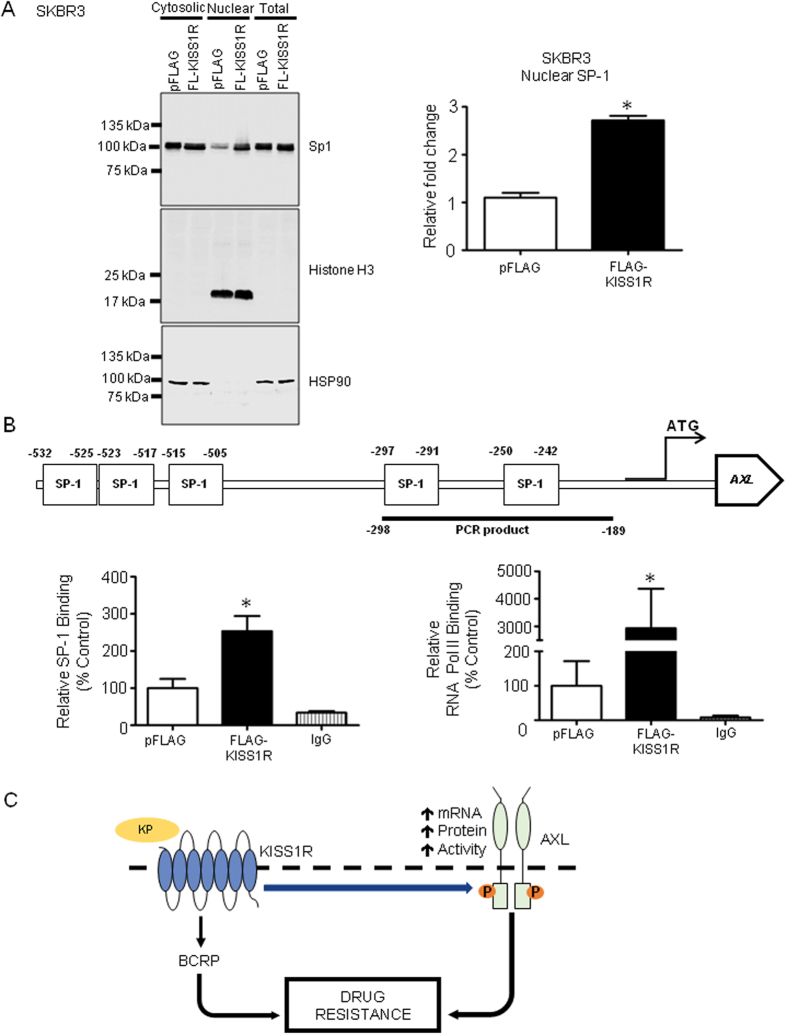
KISS1R increases SP-1-dependent *AXL* gene expression. (**A**) Western blot showing increased expression of SP-1 in the nuclear fraction of SKBR3FLAG-KISS1R cells compared to controls. Mouse anti-Histone H3 and rabbit anti-HSP90 were used as nuclear and cytosolic markers, respectively. Graph: quantification of blots; columns represent mean protein expression ± SEM. Student’s unpaired T-test: *P < 0.05; densitometric analysis of Western blot expression normalized to Histone H3 (n = 3). (**B**) Schematic showing Sp1 binding sites on the *AXL* promoter. SP-1 and RNA Pol II binding to the *AXL* promoter for the ChIP assay. Relative binding of SP-1 or RNA Pol II is expressed as a percentage of vector control binding. One SKBR3pFLAG control was arbitrarily defined as 100% (n = 4). One-way ANOVA followed by Dunnet’s multiple comparison test: *P < 0.05. Columns represent mean relative SP-1, or RNA Pol II binding ± SEM and matched IgG. (**C**) Proposed model of KISS1R induced drug resistance in TNBC: KISS1R signaling induces TNBC drug resistance by increasing the expression of drug efflux transporter BCRP expression and also via the RTK AXL, a key regulator of EMT and cell survival.

Our data reveal that KISS1R signaling regulates *AXL* as well as *BCRP* mRNA levels. The *AXL* and *BCRP* promoters have been shown to be regulated by SP-1, a transcription factor that is ubiquitously expressed in mammalian cells^53^. In fact, SP-1 is a key regulator of constitutive *AXL*^43^ as well as *BCRP* gene expression and the chemoresistance phenotype^42^. Sub-cellular fractionation of SKBR3FLAG-KISS1R cells and controls revealed that there was an enrichment of SP-1 in the nuclear fraction of cells overexpressing KISS1R compared to the controls (Fig. 8A). Histone H3 and HSP90 were used as nuclear and cytosolic markers, respectively^54^,^55^. The binding of SP-1 to the endogenous *AXL* (Fig. 8B) and *BCRP* (Supplementary Fig. 7) promoters was analyzed using ChIP assay. We observed an increase in both SP-1 binding and the recruitment of RNA Pol II to the SP-1 distal sites of the *AXL* promoter in SKBR3FLAG-KISS1R cells, compared to controls (Fig. 8B), suggesting that KISS1R regulates AXL gene transcription via SP-1. However, we did not observe a difference in SP-1 binding to the *BCRP* promoter in SKBR3FLAG-KISS1R cells, compared to controls (Supplementary Fig. 7) suggesting that KISS1R regulates *BCRP* expression by other mechanisms. The non-specific binding of IgG was tested and found to be minimal (Ct value >34). Overall, our results suggests that KISS1/KISS1R system promotes TNBC drug resistance by two mechanisms, signaling via the efflux drug transporter, BCRP and also by promoting AXL expression and activity (Fig. 8C).

## Discussion

The development of drug resistance remains a major impediment to successful treatment of cancer, specifically in patients whose primary tumour or distant metastases cannot be fully removed surgically. Uncovering the molecular mechanisms that drive tumour drug resistance is essential for developing targeted therapies that can disrupt resistance and restore drug sensitivity. Although a clear role for KISS1R signaling in promoting breast cancer progression and metastatic potential has emerged^16^,^18^,^20^,^30^, a role for KISS1R signaling in breast cancer drug resistance was not previously explored. In this report, we demonstrate for the first time that KISS1 and KISS1R are expressed in primary human TNBC tumors, and furthermore, KISS1R mRNA and protein are upregulated in TNBC patients compated to normal subjects. Similarly, *KISS1* mRNA level was also upregulated in TNBC patients, however, we could not determine whether this resulted in increased KISS1 levels in TNBC tumors by western blot analysis. Failure to detect KISS1 likely reflects its secretion from TNBC tumors since we did detect KISS1 expression in puncta in TNBC patient tumors by immunofluorescence.

We also show that KISS1R confers drug resistance using multiple ERα-negative cell lines and that KISS1R activity is necessary to promote drug resistance in these cells, since chemosensitivity was restored in the presence of the KISS1R antagonist. Stimulating cells with the KISS1R agonist KP-10, did not further increase drug resistance (data not shown), since kisspeptins are produced by these breast cancer cells, as we have previously reported^16^.

We found that KISS1R regulated the expression of the drug efflux transporter BCRP, a major regulator of the multi-drug resistance phenotype in breast cancer that is highly upregulated in TNBC^34^,^51^, and demonstrated that KISS1R-induced drug resistance was dependent on the activity of ABC transporters. BCRP appears to play a major role in this process since we did not observe a change in for the expression of *P-gp, MRP1* and *MRP4* efflux transporters, upon KISS1R overexpression in SKBR3 cells (data not shown). Although the transcription factor SP-1 is a key regulator of BCRP expression and BCRP-mediated drug resistance, KISS1R does not appear to regulate SP-1 dependent BCRP expression. Since Poll II binding to the BCRP promoter was not influenced by KISS1R, increased BCRP mRNA levels may be due to KISS1R-related changes to BCRP mRNA stability. Alternatively, Pol II recruitment could occur in another region of the BCRP promoter. BCRP transcription can be regulated by several transcription factors including AP-1, hypoxia inducible factor-1α (HIF1-α), NF-kB and nuclear EGFR binding to the BCRP promoter^51^ which might be potential mechanisms by which KISS1R signaling regulates BCRP expression.

Two of the most dysregulated signaling cascades in human carcinomas that are linked to to drug resistance^52^ as well as cell proliferation and survival are the PI3K/AKT and Ras/ERK pathways. KISS1R signaling in the drug-resistant cells resulted in an increase in the expression of anti-apoptotic proteins including AKT, ERK and survivin. Survivin, a member of the Inhibitors of Apoptosis Proteins family, is overexpressed in numerous tumours, including breast^56^. Patients who exhibit increased survivin expression are associated with shorter survival and increased resistance to therapies^56^. Interestingly, survivin has also been shown to promote BCRP expression in breast cancer cells^57^. Additionally, BCRP function^51^ and survivin expression^58^ can also be regulated by AKT. Thus it is possible that KISS1R promotes BCRP expression and function via survivin and AKT-dependent pathways.

We also identified AXL as a signaling partner in the KISS1R pathway, and demonstrated that KISS1R signaling induced AXL expression and activity. AXL overexpression correlates with poor TNBC patient prognosis^36^ and has been shown to promote breast cancer drug resistance^37^. In fact, AXL inhibitors are now under clinical investigation to treat many cancers including metastatic breast cancer^44^. AXL has also been shown to promote resistance to anti-EGFR therapies^32^. Interestingly, EGFR can bind and activate AXL independently of the AXL ligand, GAS6, in TNBC MDA-MB-231 and Hs578T cells^38^. We have previously shown that KISS1R signaling stimulated the activation of EGFR^16^,^18^ and our future studies will investigate a role for EGFR in regulating KISS1R induced drug resistance. It is noteworthy that AXL is a key regulator of EMT and cell invasion. EMT is a major driver of drug resistance, by regulating drug efflux transporter expression and activity, and increasing breast cancer cell stem-like properties^59^. We have previously shown that KISS1R signaling promotes an EMT-like phenotype^18^. Thus, KISS1R may promote drug resistance by up-regulating AXL expression, leading to increased signaling via AKT. Surprisingly, although AXL-depleted TNBC cells displayed a restoration in chemosensitivity similar to what was observed in cells treated with the KISS1R antagonist, we did not observe a change in BCRP expression upon AXL knock-down. This suggests that KISS1R signaling regulates drug resistance by regulating BCRP expression possibly via an AXL/EMT-dependent mechanism (Fig. 6C). Our observations are supported by Li and colleagues, who demonstrated that depletion of AXL in MDA-MB-231 cells decreased PI3K/AKT signaling, resulting in decreased of slug expression; this in turn suppressed breast cancer cell invasion and restored drug sensitivity^37^.

In summary, we provide evidence that KISS1R is a key regulator of drug resistance, and demonstrate KISS1, KISS1R, AXL and BCRP expression is elevated in TNBC primary tumors. Interestingly, *KISS1* mRNA expression correlated positively with *AXL* expression in these TNBC patients. Due to the current poor prognosis of TNBC patients, discovering new targets that can improve treatment and reduce resistance to chemotherapy is in urgent need. Since KISS1R signaling is now implicated in the promotion of metastasis and drug resistance, two major factors leading to poor breast cancer patient prognosis, it may be an attractive drug target for the development of therapeutics to improve treatment of TNBC patients.

## Additional Information

**How to cite this article**: Blake, A. *et al*. G protein-coupled KISS1 receptor is overexpressed in triple negative breast cancer and promotes drug resistance. *Sci. Rep.* **7**, 46525; doi: 10.1038/srep46525 (2017).

**Publisher's note:** Springer Nature remains neutral with regard to jurisdictional claims in published maps and institutional affiliations.

## Supplementary Material

Supplementary Figures

Supplementary video

## Figures and Tables

**Table 1:** Profile of Triple Negative Breast Cancer (TNBC) Patients and Normal Subjects (from Origene).

Parameter	Number (range)	%
Age (years)		
Normal Subjects	Mean 57.6 (42–82)	
TNBC	Mean 60.6 (27–84)	
Tumor size (cm)	Mean 3.4 (0.7–10)	
Hormone receptors (by IHC and FISH)		
ER negative	20/20	
PR negative	20/20	
HER2 negative	20/20	
Histology		
Adenocarcinoma of breast, ductal	20/20	
Histology Grade		
2	2	10%
3	18	90%
Stage		
IA	3	15%
IB	2	10%
IIA	9	45%
IIB	1	5%
IIIA	3	15%
IIIC	1	5%
IV	1	5%
Tumor size		
T1	7	
T2	10	
T3	3	
Node Status		
N0	11	
N1	6	
N2	1	
N3	1	
Nx	1	

**Table 2:** Profile of Triple Negative Breast Cancer (TNBC) Patients from London Health Science Centre.

Parameter	Number (range)	%
Age (years)		
Normal Subjects	Mean 45 (31–67)	
TNBC	Mean 48 (20–72)	
Tumor size (cm)	Mean 2.8 (0.7–7)	
Hormone receptors (by IHC and FISH)		
ER negative	20/20	
PR negative	20/20	
HER2 negative	20/20	
Histology		
Adenocarcinoma of breast, ductal	20/20	
Stage		
I	5	25
IIA	5	25
IIB	4	20
IIIA	1	5
IIIB	1	5
IIIC	4	20

## References

[b1] Ferlay J. . Cancer incidence and mortality worldwide: sources, methods and major patterns in GLOBOCAN 2012. International journal of cancer 136, E359–386 (2015). 25220842 10.1002/ijc.29210

[b2] Foulkes W. D., Smith I. E. & Reis-Filho J. S. Triple-negative breast cancer. The New England journal of medicine 363, 1938–1948, doi: 10.1056/NEJMra1001389 (2010). 21067385

[b3] Amiri-Kordestani L., Basseville A., Kurdziel K., Fojo A. T. & Bates S. E. Targeting MDR in breast and lung cancer: discriminating its potential importance from the failure of drug resistance reversal studies. Drug resistance updates: reviews and commentaries in antimicrobial and anticancer chemotherapy 15, 50–61, doi: 10.1016/j.drup.2012.02.002 (2012). 22464282 PMC3680361

[b4] Crown J., O’Shaughnessy J. & Gullo G. Emerging targeted therapies in triple-negative breast cancer. Annals of oncology: official journal of the European Society for Medical Oncology/ESMO 23 Suppl 6, vi56–65, doi: 10.1093/annonc/mds196 (2012). 23012305

[b5] Andre F. & Zielinski C. C. Optimal strategies for the treatment of metastatic triple-negative breast cancer with currently approved agents. Annals of oncology: official journal of the European Society for Medical Oncology/ESMO 23 Suppl 6, vi46–51, doi: 10.1093/annonc/mds195 (2012). 23012302

[b6] Dorsam R. T. & Gutkind J. S. G-protein-coupled receptors and cancer. Nature reviews. Cancer 7, 79–94, doi: 10.1038/nrc2069 (2007). 17251915

[b7] Kirby H. R., Maguire J. J., Colledge W. H. & Davenport A. P. International Union of Basic and Clinical Pharmacology. LXXVII. Kisspeptin receptor nomenclature, distribution, and function. Pharmacol Rev 62, 565–578 (2010). 21079036 10.1124/pr.110.002774 PMC2993257

[b8] Bhattacharya M. & Babwah A. V. Kisspeptin: Beyond the Brain. Endocrinologyen20141915, doi: 10.1210/en.2014-1915 (2015). 25590245

[b9] Babwah A. V. . GnRH Neuron-Specific Ablation of Galphaq/11 Results in Only Partial Inactivation of the Neuroendocrine-Reproductive Axis in Both Male and Female Mice: *In Vivo* Evidence for Kiss1r-Coupled Galphaq/11-Independent GnRH Secretion. The Journal of neuroscience: the official journal of the Society for Neuroscience 35, 12903–12916, doi: 10.1523/JNEUROSCI.0041-15.2015 (2015). 26377475 PMC4571609

[b10] Kotani M. . The metastasis suppressor gene KiSS-1 encodes kisspeptins, the natural ligands of the orphan G protein-coupled receptor GPR54. The Journal of biological chemistry 276, 34631–34636, doi: 10.1074/jbc.M104847200 (2001). 11457843

[b11] Ohtaki T. . Metastasis suppressor gene KiSS-1 encodes peptide ligand of a G-protein-coupled receptor. Nature 411, 613–617, doi: 10.1038/35079135 (2001). 11385580

[b12] Bianco S. D. . KISS1R intracellular trafficking and degradation: effect of the Arg386Pro disease-associated mutation. Endocrinology 152, 1616–1626, doi: 10.1210/en.2010-0903 (2011). 21285314 PMC3060635

[b13] Min L. . Dynamic kisspeptin receptor trafficking modulates kisspeptin-mediated calcium signaling. Mol Endocrinol 28, 16–27, doi: 10.1210/me.2013-1165 (2014). 24295737 PMC3874457

[b14] George J. T. . Kisspeptin-10 is a potent stimulator of LH and increases pulse frequency in men. The Journal of clinical endocrinology and metabolism 96, E1228–1236, doi: 10.1210/jc.2011-0089 (2011). 21632807 PMC3380939

[b15] Young J. . Kisspeptin restores pulsatile LH secretion in patients with neurokinin B signaling deficiencies: physiological, pathophysiological and therapeutic implications. Neuroendocrinology 97, 193–202, doi: 10.1159/000336376 (2013). 22377698 PMC3902960

[b16] Zajac M. . GPR54 (KISS1R) transactivates EGFR to promote breast cancer cell invasiveness. PloS one 6, e21599 (2011). 21738726 10.1371/journal.pone.0021599 PMC3125256

[b17] Pampillo M. . Regulation of GPR54 signaling by GRK2 and {beta}-arrestin. Mol Endocrinol 23, 2060–2074, doi: 10.1210/me.2009-0013 (2009). 19846537 PMC5419131

[b18] Cvetkovic D. . KISS1R induces invasiveness of estrogen receptor-negative human mammary epithelial and breast cancer cells. Endocrinology 154, 1999–2014, doi: 10.1210/en.2012-2164 (2013). 23525242

[b19] Marot, D. . High tumoral levels of Kiss1 and G-protein-coupled receptor 54 expression are correlated with poor prognosis of estrogen receptor-positive breast tumors. Endocrine-related cancer 14, 691–702 (2007). 17914099 10.1677/ERC-07-0012

[b20] Cvetkovic D., Babwah A. V. & Bhattacharya M. Kisspeptin/KISS1R System in Breast Cancer. Journal of Cancer 4, 653–661, doi: 10.7150/jca.7626 (2013). 24155777 PMC3805993

[b21] Prentice L. M. . Kisspeptin and GPR54 immunoreactivity in a cohort of 518 patients defines favourable prognosis and clear cell subtype in ovarian carcinoma. BMC medicine 5, 33, doi: 10.1186/1741-7015-5-33 (2007). 18005407 PMC2200658

[b22] Ikeguchi M., Yamaguchi K. & Kaibara N. Clinical significance of the loss of KiSS-1 and orphan G-protein-coupled receptor (hOT7T175) gene expression in esophageal squamous cell carcinoma. Clinical cancer research: an official journal of the American Association for Cancer Research 10, 1379–1383 (2004). 14977840 10.1158/1078-0432.ccr-1519-02

[b23] Guan-Zhen Y. . Reduced protein expression of metastasis-related genes (nm23, KISS1, KAI1 and p53) in lymph node and liver metastases of gastric cancer. International journal of experimental pathology 88, 175–183, doi: 10.1111/j.1365-2613.2006.00510.x (2007). 17504447 PMC2517304

[b24] Schmid K. . KiSS-1 overexpression as an independent prognostic marker in hepatocellular carcinoma: an immunohistochemical study. Virchows Archiv: an international journal of pathology 450, 143–149, doi: 10.1007/s00428-006-0352-9 (2007). 17216189

[b25] Goertzen C. G., Dragan M., Turley E., Babwah A. V. & Bhattacharya M. KISS1R signaling promotes invadopodia formation in human breast cancer cell via beta-arrestin2/ERK. Cellular signalling 28, 165–176 (2016). 26721186 10.1016/j.cellsig.2015.12.010

[b26] Smith J. T., Cunningham M. J., Rissman E. F., Clifton D. K. & Steiner R. A. Regulation of Kiss1 gene expression in the brain of the female mouse. Endocrinology 146, 3686–3692 (2005). 15919741 10.1210/en.2005-0488

[b27] Martin T. A., Watkins G. & Jiang W. G. KiSS-1 expression in human breast cancer. Clin Exp Metastasis 22, 503–511 (2005). 16320113 10.1007/s10585-005-4180-0

[b28] Papaoiconomou E. . Kiss-1/GPR54 protein expression in breast cancer. Anticancer research 34, 1401–1407 (2014). 24596387

[b29] Jarzabek K., Koda M., Kozlowski L., Milewski R. & Wolczynski S. Immunohistochemical study of KiSS1 and KiSS1R expression in human primary breast cancer: Association with breast cancer receptor status, proliferation markers and clinicopathological features. Histology and histopathology 30, 715–723 (2015). 25535062 10.14670/HH-30.715

[b30] Cho S. G. . Haploinsufficiency in the prometastasis Kiss1 receptor Gpr54 delays breast tumor initiation, progression, and lung metastasis. Cancer research 71, 6535–6546, doi: 10.1158/0008-5472.CAN-11-0329 (2011). 21852382 PMC4949591

[b31] Wang Y., Cao S. & Chen Y. Molecular Treatment of Different Breast Cancers. Anti-cancer agents in medicinal chemistry(2015). 10.2174/1871520615666150129211901 25634449

[b32] Fletcher J. I., Haber M., Henderson M. J. & Norris M. D. ABC transporters in cancer: more than just drug efflux pumps. Nature reviews. Cancer 10, 147–156 (2010). 20075923 10.1038/nrc2789

[b33] Jaeger W. Classical resistance mechanisms. International journal of clinical pharmacology and therapeutics 47, 46–48 (2009). 19203536

[b34] Britton K. M. . Breast cancer, side population cells and ABCG2 expression. Cancer letters 323, 97–105 (2012). 22521545 10.1016/j.canlet.2012.03.041 PMC3880937

[b35] O’Bryan J. P. . axl, a transforming gene isolated from primary human myeloid leukemia cells, encodes a novel receptor tyrosine kinase. Molecular and cellular biology 11, 5016–5031 (1991). 1656220 10.1128/mcb.11.10.5016-5031.1991 PMC361494

[b36] Wu X. . Global phosphotyrosine survey in triple-negative breast cancer reveals activation of multiple tyrosine kinase signaling pathways. Oncotarget 6, 29143–29160 (2015). 26356563 10.18632/oncotarget.5020 PMC4745717

[b37] Li Y. . Axl mediates tumor invasion and chemosensitivity through PI3K/Akt signaling pathway and is transcriptionally regulated by slug in breast carcinoma. IUBMB life 66, 507–518 (2014). 24984960 10.1002/iub.1285

[b38] Meyer A. S., Miller M. A., Gertler F. B. & Lauffenburger D. A. The receptor AXL diversifies EGFR signaling and limits the response to EGFR-targeted inhibitors in triple-negative breast cancer cells. Science signaling **6**, ra66, doi: 10.1126/scisignal.2004155 (2013). PMC3947921 23921085

[b39] Li T. T. . Beta-arrestin/Ral signaling regulates lysophosphatidic acid-mediated migration and invasion of human breast tumor cells. Mol Cancer Res 7, 1064–1077 (2009). 19609003 10.1158/1541-7786.MCR-08-0578

[b40] Shen F. . Quantitation of doxorubicin uptake, efflux, and modulation of multidrug resistance (MDR) in MDR human cancer cells. The Journal of pharmacology and experimental therapeutics 324, 95–102 (2008). 17947497 10.1124/jpet.107.127704

[b41] Alemayehu M. . beta-Arrestin2 regulates lysophosphatidic acid-induced human breast tumor cell migration and invasion via Rap1 and IQGAP1. PloS one 8, e56174 (2013). 23405264 10.1371/journal.pone.0056174 PMC3566084

[b42] Yang W. J. . Transcription factors Sp1 and Sp3 regulate expression of human ABCG2 gene and chemoresistance phenotype. Molecules and cells 36, 368–375 (2013). 23996530 10.1007/s10059-013-0191-x PMC3887993

[b43] Mudduluru G. & Allgayer H. The human receptor tyrosine kinase Axl gene–promoter characterization and regulation of constitutive expression by Sp1, Sp3 and CpG methylation. Bioscience reports 28, 161–176 (2008). 18522535 10.1042/BSR20080046

[b44] Wu X. . AXL kinase as a novel target for cancer therapy. Oncotarget 5, 9546–9563 (2014). 25337673 10.18632/oncotarget.2542 PMC4259419

[b45] Schmidt M. & Lichtner R. B. EGF receptor targeting in therapy-resistant human tumors. Drug resistance updates: reviews and commentaries in antimicrobial and anticancer chemotherapy 5, 11–18 (2002). 12127860 10.1016/s1368-7646(02)00004-3

[b46] Sahin Z., Canpolat S., Ozcan M., Ozgocer T. & Kelestimur H. Kisspeptin antagonist prevents RF9-induced reproductive changes in female rats. Reproduction 149, 465–473, doi: 10.1530/REP-14-0683 (2015). 25713426

[b47] Roseweir A. K. . Discovery of potent kisspeptin antagonists delineate physiological mechanisms of gonadotropin regulation. The Journal of neuroscience: the official journal of the Society for Neuroscience 29, 3920–3929, doi: 10.1523/JNEUROSCI.5740-08.2009 (2009). 19321788 PMC3035813

[b48] Pineda R. . Critical roles of kisspeptins in female puberty and preovulatory gonadotropin surges as revealed by a novel antagonist. Endocrinology 151, 722–730, doi: 10.1210/en.2009-0803 (2010). 19952274

[b49] Rasoulzadeh Z. . Placental Kisspeptins Differentially Modulate Vital Parameters of Estrogen Receptor-Positive and -Negative Breast Cancer Cells. PloS one 11, e0153684, doi: 10.1371/journal.pone.0153684 (2016). 27101408 PMC4839747

[b50] Joensuu H. & Gligorov J. Adjuvant treatments for triple-negative breast cancers. Annals of oncology: official journal of the European Society for Medical Oncology/ESMO 23 Suppl 6, vi40–45, doi: 10.1093/annonc/mds194 (2012). 23012301

[b51] Natarajan K., Xie Y., Baer M. R. & Ross D. D. Role of breast cancer resistance protein (BCRP/ABCG2) in cancer drug resistance. Biochemical pharmacology 83, 1084–1103 (2012). 22248732 10.1016/j.bcp.2012.01.002 PMC3307098

[b52] Davis J. M. . Raf-1 and Bcl-2 induce distinct and common pathways that contribute to breast cancer drug resistance. Clinical cancer research: an official journal of the American Association for Cancer Research 9, 1161–1170 (2003). 12631622

[b53] Kingsley C. & Winoto A. Cloning of GT box-binding proteins: a novel Sp1 multigene family regulating T-cell receptor gene expression. Molecular and cellular biology 12, 4251–4261 (1992). 1341900 10.1128/mcb.12.10.4251-4261.1992 PMC360348

[b54] Marchenko N. D. . Stress-mediated nuclear stabilization of p53 is regulated by ubiquitination and importin-alpha3 binding. Cell death and differentiation 17, 255–267, doi: 10.1038/cdd.2009.173 (2010). 19927155 PMC4419752

[b55] Ahmed K. M., Fan M., Nantajit D., Cao N. & Li J. J. Cyclin D1 in low-dose radiation-induced adaptive resistance. Oncogene 27, 6738–6748, doi: 10.1038/onc.2008.265 (2008). 18695676 PMC6759063

[b56] Jha K., Shukla M. & Pandey M. Survivin expression and targeting in breast cancer. Surgical oncology 21, 125–131, doi: 10.1016/j.suronc.2011.01.001 (2012). 21334875

[b57] Wang Q. P. . Survivin up-regulates the expression of breast cancer resistance protein (BCRP) through attenuating the suppression of p53 on NF-kappaB expression in MCF-7/5-FU cells. The international journal of biochemistry & cell biology 45, 2036–2044 (2013). 23838170 10.1016/j.biocel.2013.06.026

[b58] Zhao P. . Regulation of survivin by PI3K/Akt/p70S6K1 pathway. Biochemical and biophysical research communications 395, 219–224 (2010). 20361940 10.1016/j.bbrc.2010.03.165

[b59] Mallini P., Lennard T., Kirby J. & Meeson A. Epithelial-to-mesenchymal transition: what is the impact on breast cancer stem cells and drug resistance. Cancer treatment reviews 40, 341–348, doi: 10.1016/j.ctrv.2013.09.008 (2014). 24090504

