# Supplementary material for: G protein-coupled KISS1 receptor is overexpressed in triple negative breast cancer and promotes drug resistance
Source: Sci Rep. 2017 Apr 19;7:46525. doi: 10.1038/srep46525 (PMC5395950; doi:10.1038/srep46525)

Supplementary Figure Legends

G protein-coupled KISS1 receptor is overexpressed in triple negative breast cancer and promotes drug resistance

Authors: Alexandra Blake, Magdalena Dragan, Rommel G. Tirona, Daniel B. Hardy, Muriel Brackstone, Alan B. Tuck, Andy V. Babwah, Moshmi Bhattacharya

Supplementary Movie (Scratch assay). Time-lapse microscopy of SKBR3 cells expressing pFLAG vector controls or FLAGKISS1R (top and bottom panels, respectively); images acquired every 15 minutes for 24 h using an Olympus IX-81 microscope.

Supplementary Figure 1. KISS1R and KISS1 expression in TNBC patient primary tumors and normal breast tissue. Representative western blots showing the expression of endogenous KISS1R and KISS1 in (A, B) normal healthy breast tissue and (C, D) TNBC primary tumors, compared to expression of each protein in MDA-MB-231 cell lysates (positive control). Total of 13 healthy and 20 TNBC tumors were used for analysis. Representative images showing immunofluorescence and confocal microscopy of KISS1R expression in (E) SKBR3 FLAG-KISS1R cells and (F) the localization of KISS1R in lamellipodia of motile SKBR3 FLAG-KISS1R cells; SKBR3 pFLAG (vector control) cells have weak endogenous KISS1R expression. Arrow shows the direction of cell migration into the scratch (n=5). KISS1R immunostaining detected using a rabbit polyclonal KISS1R antibody followed by donkey anti-rabbit AF488 (green), and Hoechst (blue) nuclear stain. *Scale Bar,* 20 µm.

Supplementary Figure 2. Cell Growth Assays. SKBR3 pFLAG vector controls and SKBR3 FLAG-KISS1R cells were cultured for 72 hours with or without 100 nM KP-10 and counted at 24 hour intervals (n=3). Two-way ANOVA followed by Bonferroni post-hoc test: a, $P<0.05$ for pFLAG compared to FLAGKISS1R; b, $P<0.05$ for pFLAG+KP10 when compared to FLAGKISS1R+KP10

Supplementary Figure 3. Quantification of blots shown in Figures 2 and 5. Densitometric analysis of protein expression in SKBR3FLAG-KISS1R cells and controls (from Fig. 2C) for (A) KISS1R, (B) KISS1, and cell survival molecules (C) AXL, (D) AKT, (E) ERK, (F) survivin (n=4-6). (G) Densitometric analysis of protein expression for BCRP in SKBR3FLAG-KISS1R cells and controls shown in Fig. 5D (n=5). In each case, protein expression was normalized to β-actin. Columns represent mean protein expression ± SEM. Student's unpaired T-test: *, $P<0.05$.

Supplementary Figure 4. Quantification of AXL phosphorylation and expression. (A) Quantification of phosphorylated AXL expression normalized to total AXL expression from Fig. 6A (n=4). (B) Densitometric analysis of blots showing AXL protein expression in Fig. 6B (n=5). (C) Densitometric analysis of blots from Fig. 7A showing AXL protein expression in SKBR3FLAG-KISS1R cells expressing AXL siRNA and scrambled controls. (D) Densitometric analysis of blots from Fig. 7D showing AXL protein expression in TNBC MDA-MB-231 cells expressing AXL siRNA and scrambled controls. Densitometric analysis of blots from Fig. 7G showing protein expression of (E) Snail-Slug, (F) BCRP in SKBR3FLAG-KISS1R cells expressing AXL siRNA and scrambled controls (n=4). Densitometric analysis of blots from Fig.

7H showing protein expression of (G) N-cadherin, (H) BCRP in MDA-MB-231 cells expressing AXL siRNA and scrambled controls (n=3). Columns represent mean protein expression ± SEM. One-way ANOVA followed by Dunnet's multiple comparison test or Student's unpaired T-test: *, $P<0.05$.

Supplementary Figure 5. Protein expression in mammary cell lysates. KISS1, KISS1R and AXL expression in breast cancer cell lines . Note, T47D, MCF7: ERα-positive breast cancer cell lines; n=4. Columns represent mean protein expression ± SEM. One-way ANOVA followed by Dunnet's multiple comparison test: a, $P<0.05$ SKBR3FLAG-KISS1R compared to SKBR3 pFLAG; b, $P<0.05$ SKBR3 FLAG-KISS1R compared to T47D; c, $P<0.05$ SKBR3 FLAG-KISS1R compared to MCF7; d, $P<0.05$ SKBR3FLAG-KISS1R compared to MDA-MB-231; e, $P<0.05$ MDA-MB-231 compared to SKBR3pFLAG; f, $P<0.05$ MDA-MB-231 compared to T47D; g, $P<0.05$ MDA-MB-231 compared to MCF7.

Supplementary Figure 6. AXL depletion does not regulate expression levels of BCRP. (A) Representative Western blot showing protein levels of AXL and BCRP in SKBR3FLAG-KISS1R cells expressing AXL siRNA and scrambled controls, 72 and 96 h after transfection (n=3); densitometric analysis of blots shown on the right. Columns represent mean protein expression ± SEM. Student's unpaired T-test: *, $P<0.05$ vs SControl for each time point.

Supplementary Figure 7. KISS1R does not regulate SP-1-dependent *BCRP* gene expression. Schematic showing Sp1 binding sites on the *BCRP* promoter. SP-1 and RNA Pol II binding to the *BCRP* promoter by ChIP analysis using an anti SP-1-specific or RNA Pol II-specific antibodies. Relative binding of SP-1 or RNA Pol II is expressed as a percentage of vector control binding. One SKBR3 pFLAG control was arbitrarily defined as 100% (n=4). One-way ANOVA followed by Dunnet's multiple comparison test: *, P<0.05. Columns represent mean relative SP-1 or RNA Pol II binding ± SEM.

Supplementary Figure 8. Representative full Western blots from Figures 2-7. (A-F) blots from Figure 2C; (G, H) blots from Figure 4 D, E; (I) blots from Figure 5D; (J) blots from Figure 6A; (K) blot from figure 7A; (L) blot from Figure 7D; (M, N) blot from Figure 7G; (O) blot from Figure 7H.

Supplementary Figure 1 Blake et al.

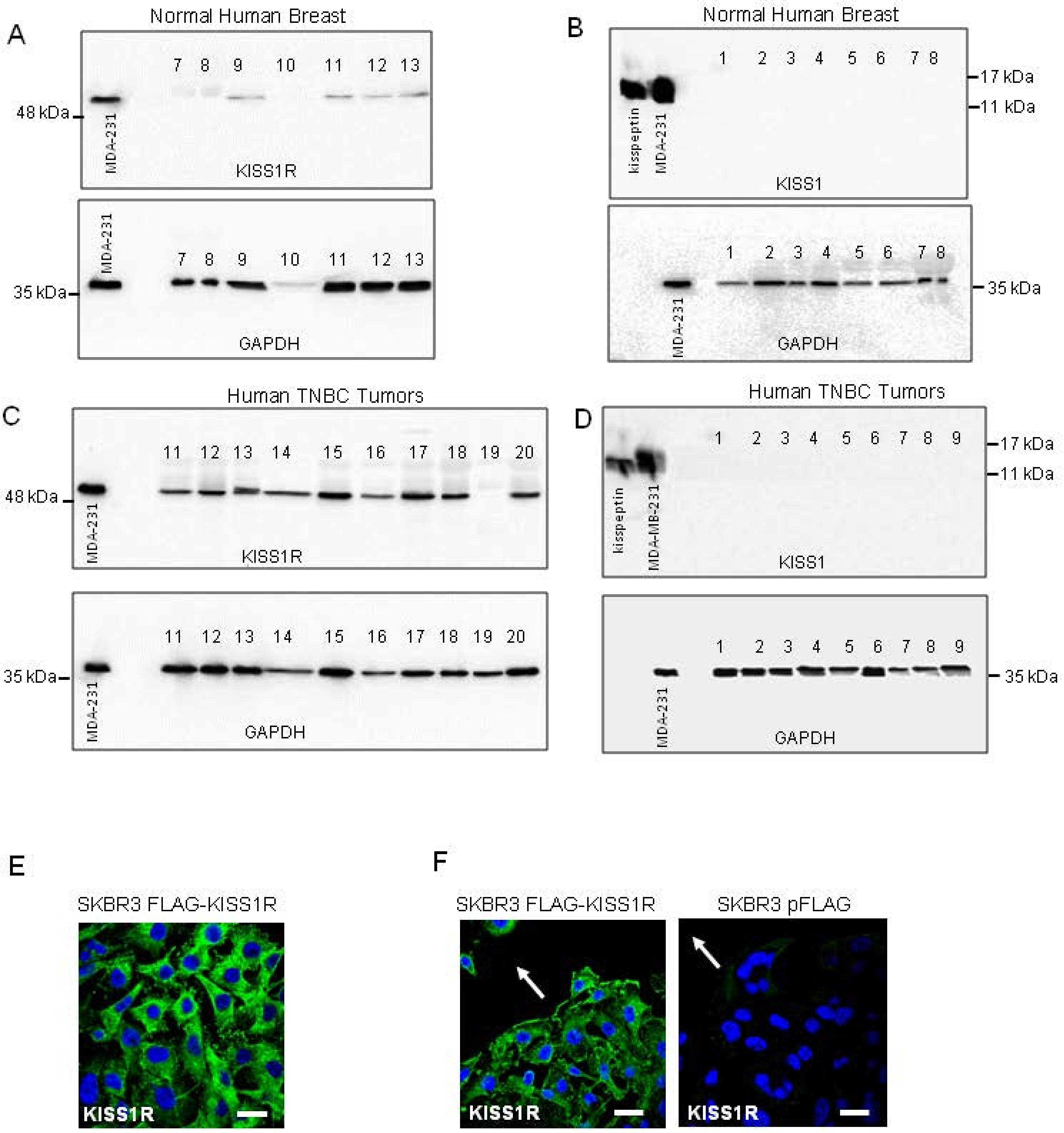

Supplementary Figure 2 Blake et al.

A

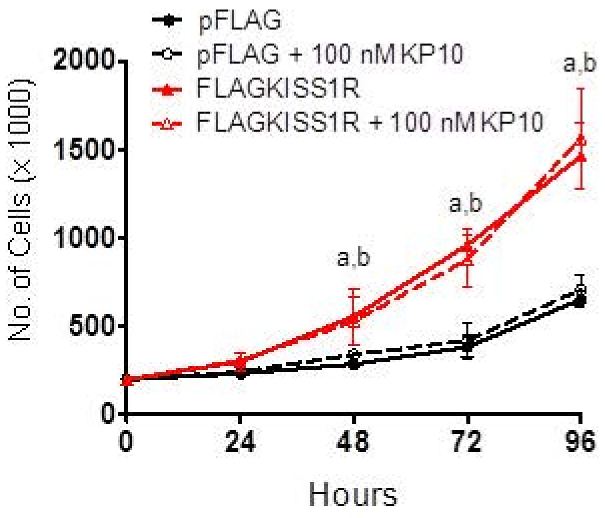

Supplementary Figure 3 Blake et al.

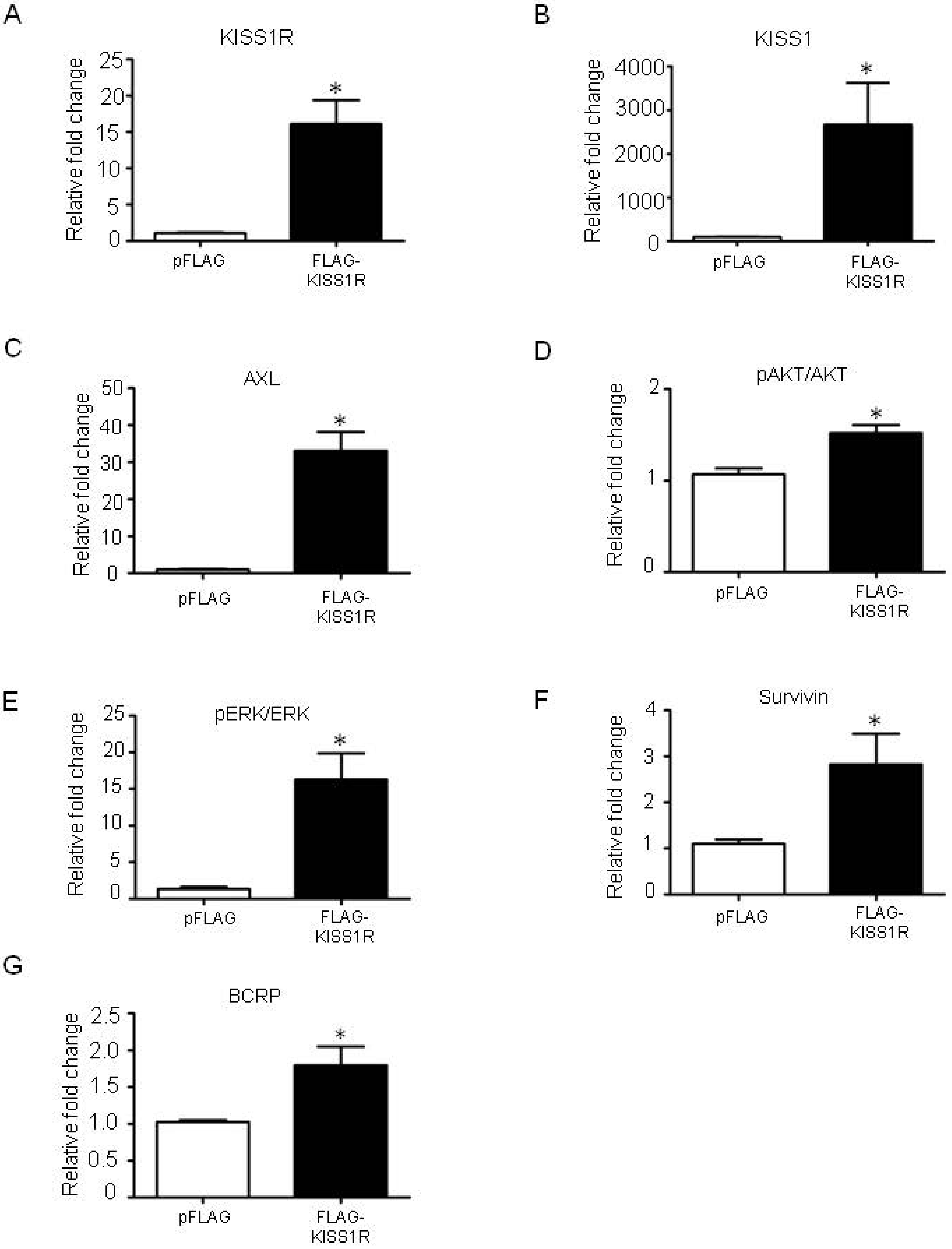

Supplementary Figure 4 Blake et al.

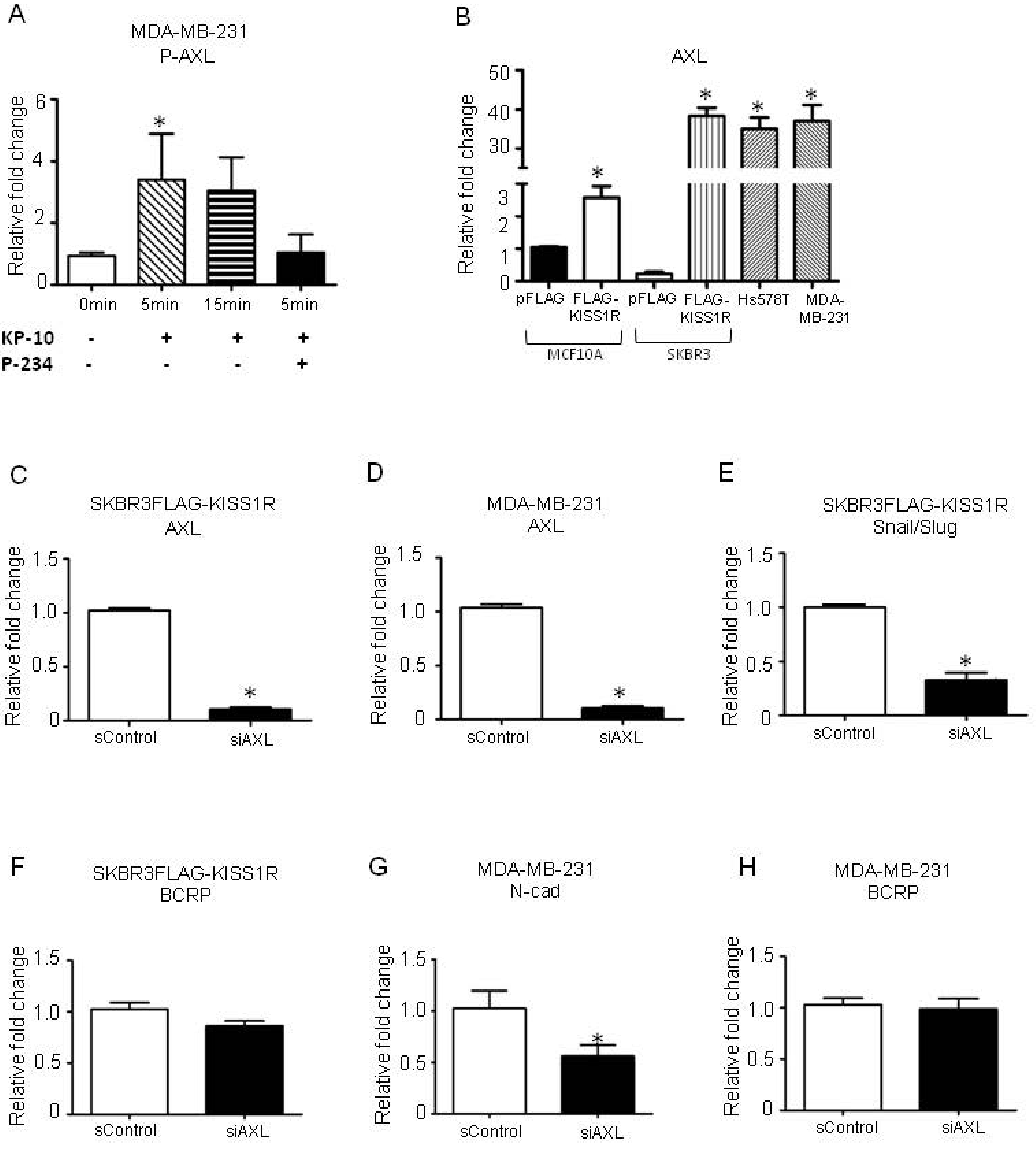

Supplementary Figure 5 Blake et al.

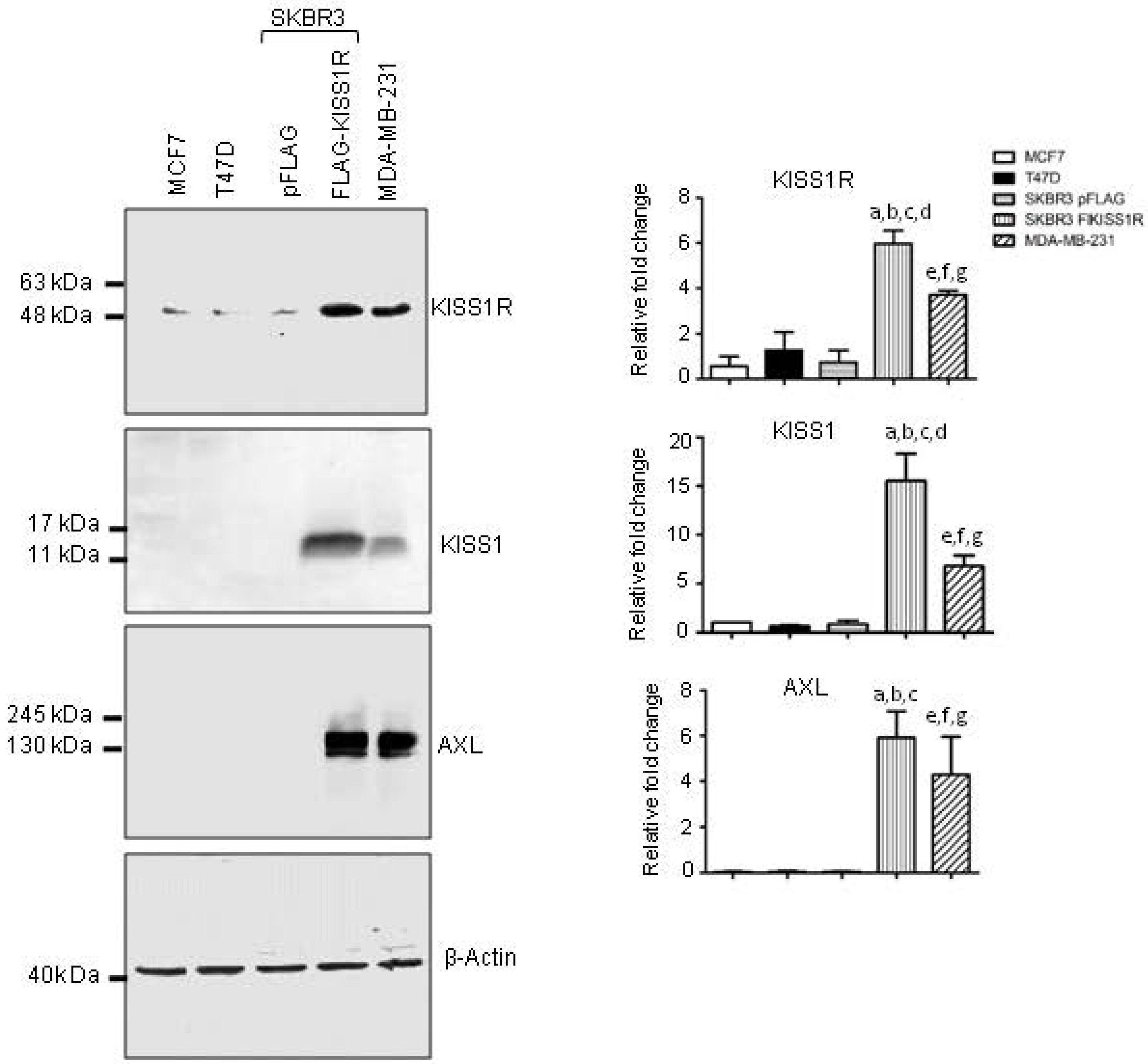

Supplementary Figure 6 Blake et al.

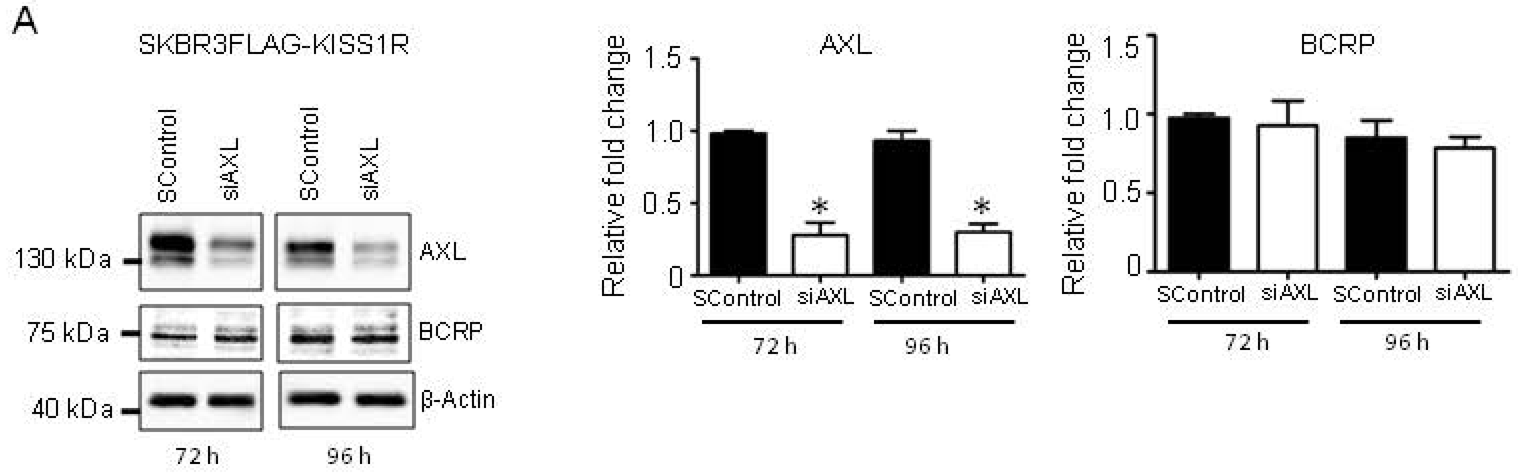

Supplementary Figure 7 Blake et al.

A

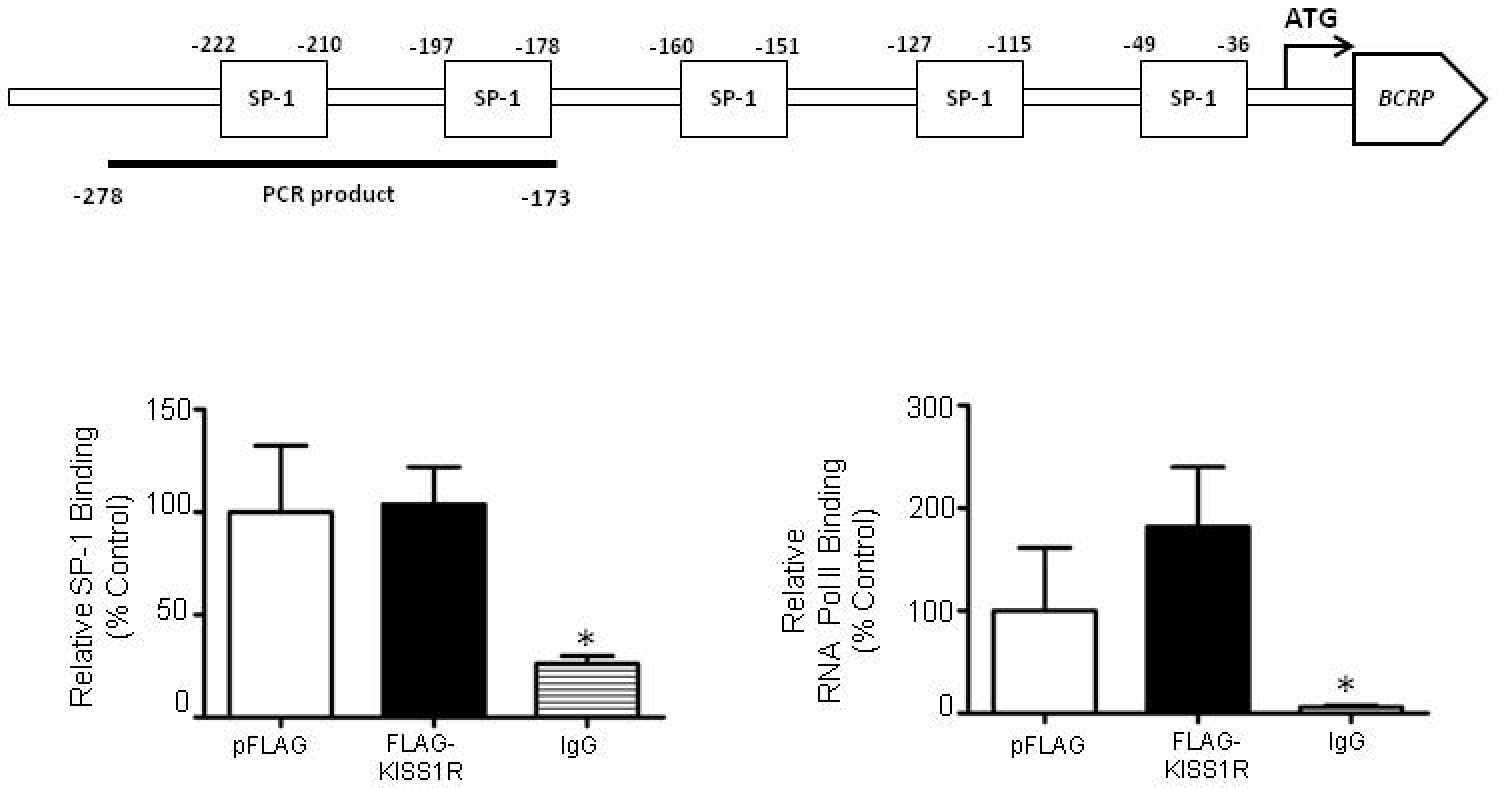

Supplementary Figure 8 Blake et al.

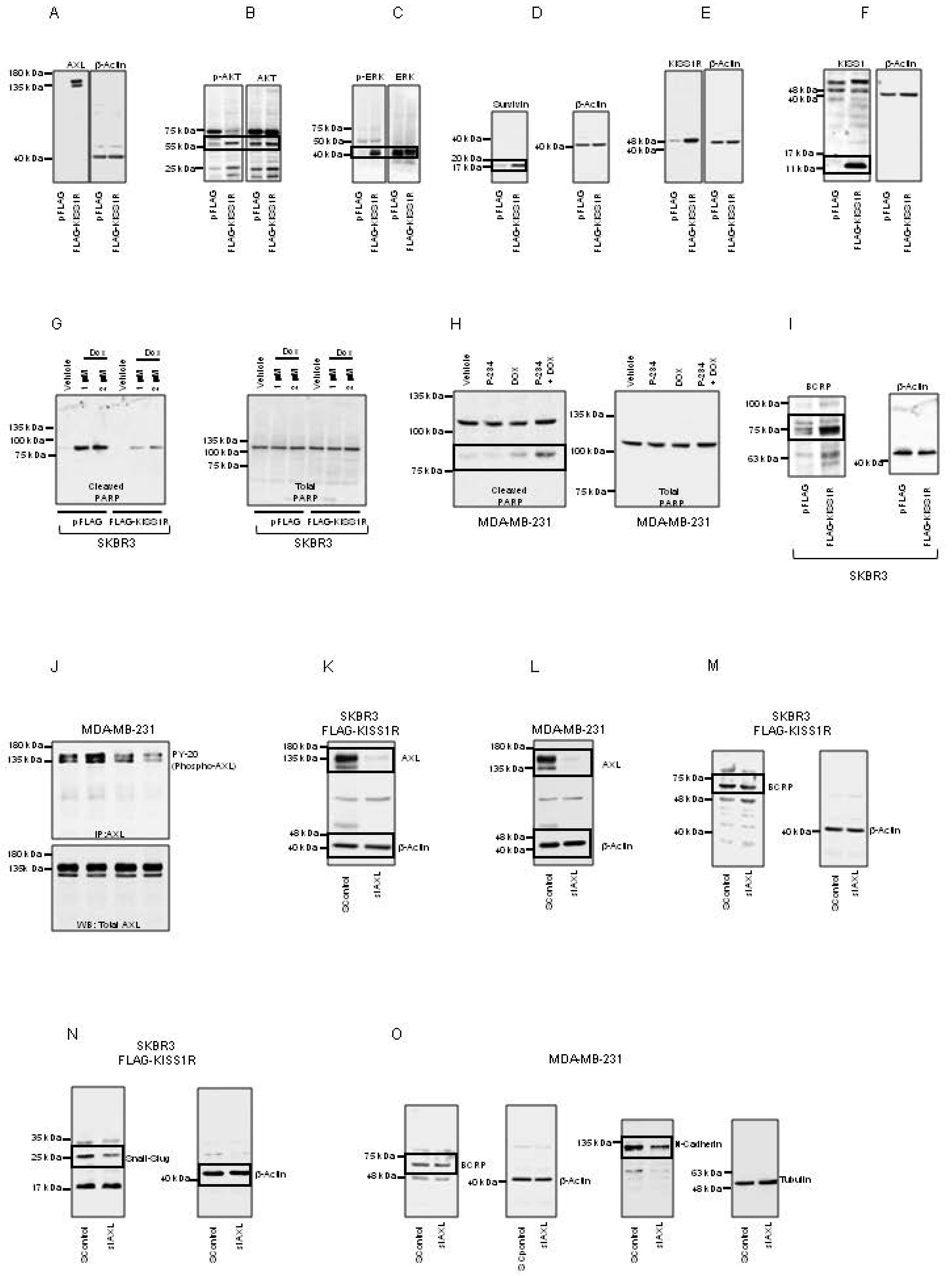

Supplement: Supplementary Figures [file srep46525-s1.pdf]
